# Down-regulation of CK2α correlates with decreased expression levels of DNA replication minichromosome maintenance protein complex (MCM) genes

**DOI:** 10.1038/s41598-019-51056-5

**Published:** 2019-10-10

**Authors:** Susanne Schaefer, Thomas K. Doktor, Sabrina B. Frederiksen, Kathleen Chea, Mirka Hlavacova, Gitte H. Bruun, Maj Rabjerg, Brage S. Andresen, Isabel Dominguez, Barbara Guerra

**Affiliations:** 1 Department of Biochemistry and Molecular Biology and the Villum Center for Bioanalytical Sciences, University of Southern Denmark, Odense, Denmark; 2 Department of Pathology, Aalborg University Hospital, Aalborg, Denmark; 3 Department of Medicine, Boston University School of Medicine, Boston, 02118 MA USA

**Keywords:** Biological techniques, Cell biology

## Abstract

Protein kinase CK2 is a serine/threonine kinase composed of two catalytic subunits (CK2α and/or CK2α’) and two regulatory subunits (CK2β). It is implicated in every stage of the cell cycle and in the regulation of various intracellular pathways associated with health and disease states. The catalytic subunits have similar biochemical activity, however, their functions may differ significantly in cells and *in vivo*. In this regard, homozygous deletion of *CK2α* leads to embryonic lethality in mid-gestation potentially due to severely impaired cell proliferation. To determine the CK2α-dependent molecular mechanisms that control cell proliferation, we established a myoblast-derived cell line with inducible silencing of CK2α and carried out a comprehensive RNA-Seq analysis of gene expression. We report evidence that CK2α depletion causes delayed cell cycle progression through the S-phase and defective response to replication stress. Differential gene expression analysis revealed that the down-regulated genes were enriched in pathways implicated in cell cycle regulation, DNA replication and DNA damage repair. Interestingly, the genes coding for the minichromosome maintenance proteins (MCMs), which constitute the core of the replication origin recognition complex, were among the most significantly down-regulated genes. These findings were validated in cells and whole mouse embryos. Taken together, our study provides new evidence for a critical role of protein kinase CK2 in controlling DNA replication initiation and the expression levels of replicative DNA helicases, which ensure maintenance of proliferative potential and genome integrity in eukaryotic cells.

## Introduction

Protein kinase CK2 is a serine-threonine kinase with orthologs in all eukaryotes from plants to yeast and mammals^1^. This enzyme is evolutionarily conserved among species and has been linked to the regulation of intracellular processes including DNA transcription, protein translation and circadian rhythm and associated with various diseases particularly cancer, neurodegenerative disorders and inflammation emerging as a key cellular regulator in both health and disease states (reviewed in^2–5^). In mammals, CK2 can be expressed in the form of tetrameric holoenzyme composed of two catalytic (CK2α and/or CK2α’) and two regulatory subunits (CK2β), however, accumulating evidence from mouse models and cell lines have revealed functional specialization of the individual isoforms, challenging the traditional view of CK2 as a stable tetrameric enzyme. In this respect, numerous studies have shown that the three proteins may display different subcellular localization and expression pattern and levels, and have independent interaction partners (Refs^6,7^ and reviewed in^8,9^). In addition, CK2α is required for preserving the stability of CK2β by a mechanism involving intermolecular phosphorylation of the latter in the N-terminal domain^10^.

Data using gene targeting by homologous recombination have significantly strengthened the notion that the CK2α and CK2α’ isoforms might be functionally specialized^1^ and although the corresponding genes are highly homologous with approximately 90% sequence identity^10^ genetic studies have shown that the phenotypic response is markedly different *in vivo*^11,12^. CK2α is the more abundant catalytic isoform and with broader expression pattern. *CK2α*+/− mice have a normal lifespan and no apparent phenotype, while mice lacking *CK2α* die by embryonic day (E) 11.0 and show abnormalities in a number of tissues and organs including the heart and neural tube due to diminished cell proliferation and not increased cell death^12–14^. Conversely, homozygous deletion of *CK2α’* results in viable mice although the males are affected by oligospermia, which results in infertility^11^.

CK2 has been shown to positively regulate cell cycle progression in a number of cancer cell lines (reviewed in^2,15^) by interacting and/or phosphorylating cell cycle-regulatory proteins (e.g. p53, p21^WAF1/CIP1^, PLK1, Chk1, Wee1)^16–19^ and proteins involved in the DNA damage response (e.g. XRCC1, MDC1, DNA-PK and 53BP1)^20–24^. Even though CK2 has been linked to these proteins, to date, there is no clear evidence of this enzyme’s targets in non-cancerous cells and *in vivo*.

In order to identify the CK2α-dependent molecular events controlling cell division on a wide scale, we generated myoblasts with inducible down-regulation of CK2α and carried out a global gene expression profiling by RNA sequencing (RNA-Seq). We provide for the first time *in vitro* and *in vivo* evidence showing that lack of CK2α negatively affects important components of the DNA replication machinery uncovering a previously uncharacterized role of CK2 in the maintenance of replication fork integrity in eukaryotic cells.

## Results

### Generation and characterization of a myoblast cell line with inducible down-regulation of CK2α

**Figure 1:**
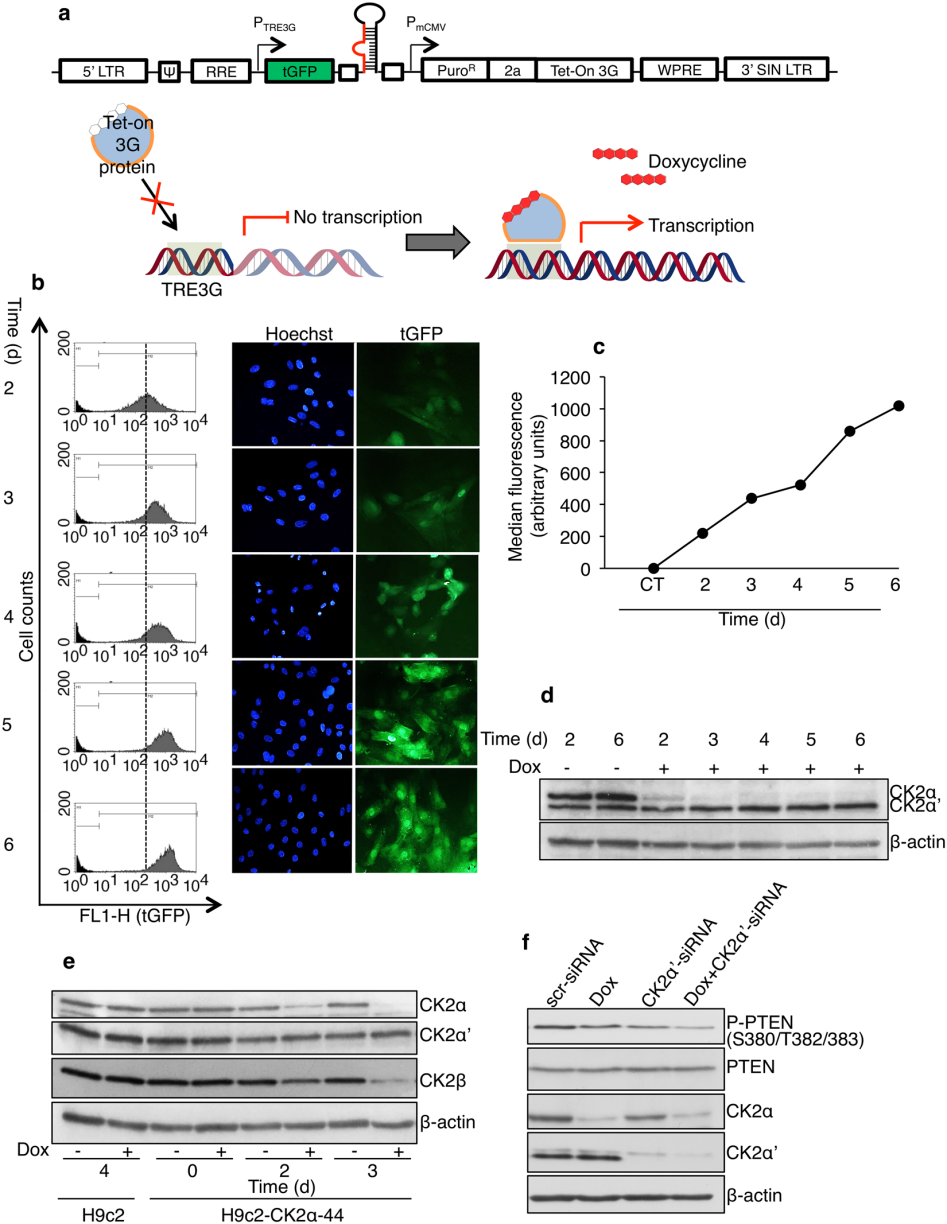
Establishment of the doxycycline-regulated H9c2-CK2α-44 cell line with inducible silencing of CK2α. (**a**) H9c-2 cells were transduced with lentiviral particles carrying a SMARTchoice inducible CK2α-shRNA construct containing a turbo-GFP (tGFP) reporter gene (upper). Expression of CK2α-shRNA is induced in the presence of doxycycline (lower). (**b**) The H9c-2-derived cell line (i.e. H9c2-CK2α-44) stably incorporating the construct was analyzed by flow cytometry in the presence of 1 µg/ml doxycycline for up to six days. Quantification of green fluorescence emission (tGFP-positive cells) indicative of the efficiency of shRNA transcription is shown in the graph. Dashed line indicates the median levels of tGFP expression after two days of incubation for both doxycycline-treated (grey peak) and control cells (black peak). Fluorescence-based pictures of cells showing increasing expression of tGFP in the presence of doxycycline are shown on the right side. Cell nuclei were visualized by Hoechst 33258 staining. (**c**) Median fluorescence is shown in the dot plot in arbitrary units. Vehicle control (CT) at six days is depicted. Two independent experiments were carried out. Results from one representative experiment are shown. (**d**) H9c2-CK2α-44 cells were treated with vehicle (i.e. dd water) or with 1 µg/ml doxycycline (Dox) for increasing amounts of time. Whole cell lysates were analyzed by Western blot employing a mouse monoclonal antibody against CK2α and CK2α’. β-actin detection served as loading control. (**e**) H9c2-CK2α-44 cells and the parental cell line were harvested after 0, 2 and 3 days of treatment with vehicle (−) or 1 µg/ml doxycycline (+) and whole cell lysates were analyzed by Western blot employing the indicated antibodies. β-actin detection served as loading control. (**f**) H9c2-CK2α-44 cells were incubated with 1 µg/ml doxycycline for three days, transfected with scr-siRNA and CK2α’-siRNA for three days, respectively, as indicated in the figure. Last lane refers to cells treated with doxycycline and transfected with CK2α’-siRNA for three days. Whole cell lysates were analyzed by Western blot employing the indicated antibodies. All experiments were performed three times obtaining similar results; one Western blot experiment of three is shown. Abbreviations: LTR: 5’ Long Terminal Repeat; Ψ: Psi packaging sequence; RRE: Rev response element; P_TRE3G_: Inducible promoter with tetracycline response elements; P_mCMV_: SMARTchoice promoter; Puro^R^: Puromycin resistance; 2a: Self-cleaving peptide; Tet-On 3G: Doxycycline-regulated transactivator protein; WPRE: Woodchuck hepatitis post-transcriptional regulatory element; 3’SIN LTR: 3’ Self-inactivating long terminal repeat.

In order to systematically examine the role of CK2α in the control of proliferation in non-cancerous cells, we created a myoblast cell line derived from H9c-2 cells with inducible down-regulation of *CK2α*. Cultured myoblasts are one of the models chosen for studying biological processes *in vitro*. The H9c-2 myoblast cell line isolated from ventricular tissue, is currently used as a mimetic for skeletal muscle but it also has the ability to differentiate towards a cardiac-like phenotype under appropriate experimental conditions responding similarly to neonatal cardiomyocytes to several stimuli^25^. We transduced H9c-2 myoblast cells with a tGFP-expressing lentiviral-based vector designed to express a short-hairpin RNA (shRNA) targeting rat *CK2α* under the control of doxycycline (Fig. 1a). Because a myoblast cell line with inducible down-regulation of CK2α had not been previously described, we first characterized biochemically the newly established cell line (hereafter referred to as H9c2-CK2α-44). To determine the timing and extent of transduction, H9c2-CK2α-44 cells were analyzed for tGFP expression following addition of doxycycline. Cells were harvested at various intervals for up to six days and green fluorescence emission was determined by flow cytometry. As indicated in Fig. 1b,c, virtually all the cells were able to express tGFP and showed increasing fluorescence signal in a time-dependent fashion indicating the successful stable transduction of the target cells. Levels of expression of CK2α were determined by Western blot. Results shown in Fig. 1d revealed high intracellular levels of CK2α than CK2α’ in the absence of doxycycline. Incubation with doxycycline for up to six days resulted in nearly complete disappearance of CK2α protein, a slight increase in the expression of CK2α’ and decreased expression levels of CK2β (Fig. 1e). To support the molecular effects of CK2α disappearance on a known intracellular CK2 target protein, we analyzed the phosphorylation status of PTEN at S380/T382/383^26^. Western blot analysis on whole lysate from cells treated as indicated in Fig. 1f revealed that the levels of phosphorylation of PTEN were decreased in cells with reduced expression of the individual CK2 catalytic isoforms as compared to control experiment (Fig. 1f, lanes 2 and 3 *vs* lane 1). PTEN phosphorylation further decreased when CK2α and CK2α’ were simultaneously down-regulated (Fig. 1f, lane 4) suggesting that both isoforms contribute to PTEN phosphorylation.

### Down-regulation of CK2α interferes with cell cycle progression and cell proliferation

**Figure 2:**
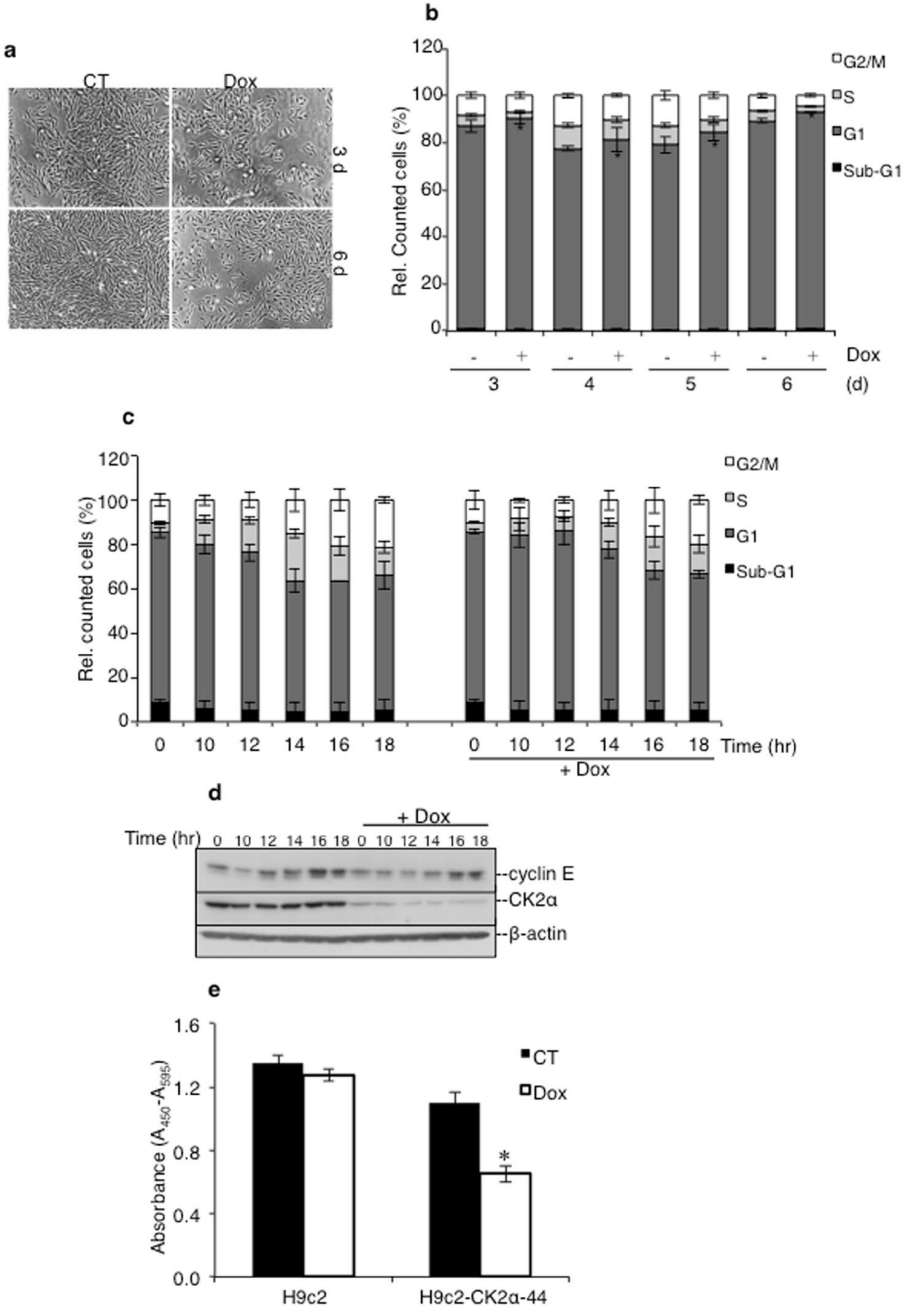
Down-regulation of CK2α perturbs G1/S cell cycle transition dynamics. (**a**) H9c2-CK2α-44 cells were treated with vehicle (CT) or doxycycline for three days and six days, respectively. Phase contrast images were taken at 50x magnification. (**b**) Cells were incubated with vehicle or 1 μg/ml doxycycline for up to six days as indicated in the figure. Cell cycle analysis was performed following propidium iodide staining. Amount of cells in the various phases of the cell cycle are expressed in percentage. *P < 0.05 with respect to control experiment. Figure shows the results of three independent experiments. (**c**) Cells were synchronized by serum starvation for 48 h in the absence or presence of doxycycline and harvested at the indicated time points after release from starvation. Cell synchronization was confirmed by flow cytometry analysis. The experiment was repeated three times obtaining similar results. (**d**) Western blot analysis of whole lysate from cells treated as indicated in (**c**) was carried out employing the indicated antibodies. Experiments were repeated three times obtaining similar results. One representative experiment is shown. (**e**) H9c-2 and H9c2-CK2α-44 cells left untreated or incubated with 1 µg/ml doxycycline for three days were labeled with BrdU during the last eight hours of incubation time. Detection of fixed cells was carried out employing an anti-BrdU antibody coupled to horseradish peroxidase. Colorimetric reactions were quantified by measuring the absorbance at 450 nm. Values are shown in arbitrary units as average of six replicates +/− STDEV, *P < 0.00001 with respect to control (i.e. cells treated with vehicle). Experiments were repeated twice obtaining similar results.

We examined the effect of *CK2α* silencing on the proliferation of H9c2-CK2α-44 cells essentially by three complementary approaches: i.e. FACS analysis, Western blot and BrdU-based assay. Incubation with doxycycline for three and six days, respectively, resulted in marked differences in the cell density as compared to control cells (Fig. 2a). Flow cytometry analysis of H9c2-CK2α-44 cells was carried out at various time points after the addition of doxycycline to determine whether the reported differences resulted from cell cycle perturbation and/or enhanced cell death (Fig. 2b). Analysis of DNA content revealed a reproducible and significant slightly increased G1 population for up to six days of incubation time with doxycycline as compared to control experiments. The fraction of cells in sub-G1, which provides an indicative measurement of cell death, was, however, negligible and could not explain the significant decrease in cell density observed in cells with lowered expression of CK2α (Fig. 2a). Next, we synchronized cells by serum starvation and looked at their ability to resume the cell cycle after adding complete growth medium. As shown in Fig. 2c, cells expressing CK2α resumed proliferation and reached the S phase after 12 h from serum deprivation as also confirmed by the analysis of the expression of cyclin E which is considered a critical regulator of the G1-S transition (Fig. 2d,^27,28^). Conversely, cells lacking CK2α did not resume the cell cycle at the same pace showing, instead, a delayed progression from G1 to S phase (Fig. 2c,d). In support of these results, cellular BrdU incorporation assay carried out with H9c2-CK2α-44 cells showed fewer cells in S-phase three days after addition of doxycycline suggesting that down-regulation of CK2α resulted in either less efficient or delayed entry into S-phase (Fig. 2e). Analysis of the parental cell line did not show any difference on BrdU incorporation under the same experimental conditions indicating that these perturbations of the cell cycle could account for the observed slow proliferation rate in cells lacking *CK2α*.

### CK2α silencing results in enhanced sensitivity to replication stress

**Figure 3:**
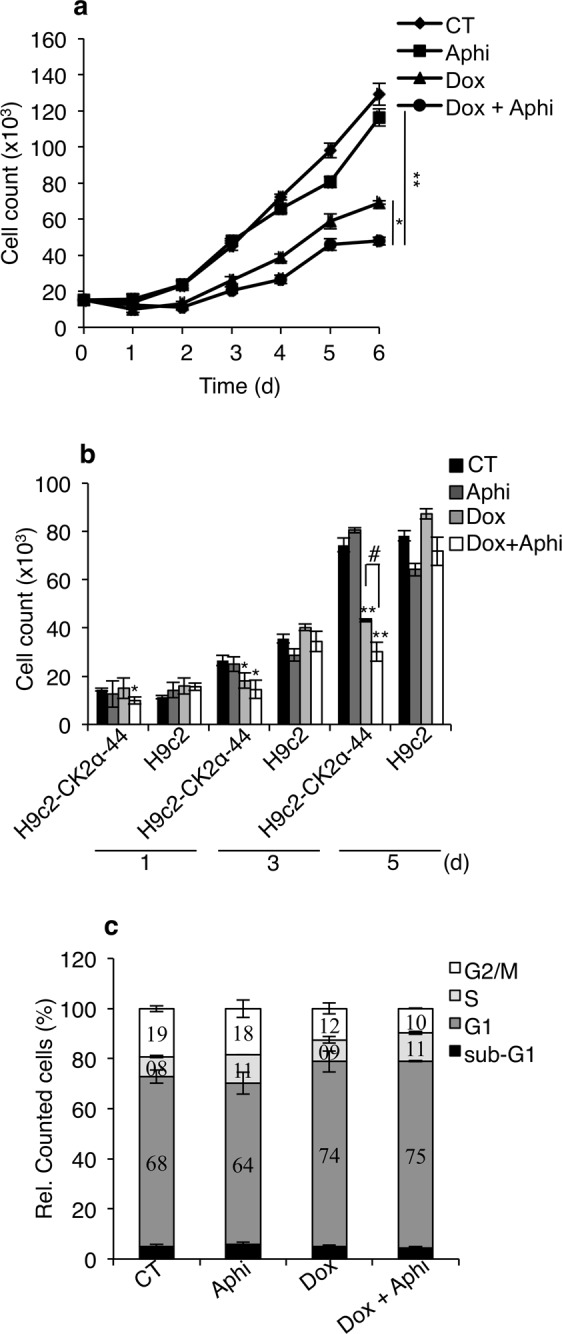
Analysis of cell proliferation following induction of mild DNA replication stress. (**a**) H9c2-CK2α-44 incubated with 1 µg/ml doxycycline for three days were subsequently re-seeded and treated with 0.1 µM aphidicolin (Aphi) for increasing amounts of time as indicated in the figure. Control experiments (CT) refer to cells grown in the presence of 0.1% DMSO for up to five days. Cell proliferation was determined by hemocytometer counting. Experiments were carried out three times in triplicates obtaining similar results. Results of one representative experiment are shown +/− STDEV, *P < 0.05, **P < 0.005. (**b**) Comparison between H9c2-CK2α-44 and H9c-2 cells with respect to proliferation efficiency. Cells were treated essentially as described in (**a**) for the indicated times. Mean values+/− STDEV of one representative experiment out of three is shown. *P < 0.05, **P < 0.0005 with respect to CT, ^#^*P* < 0.01. (**c**) H9c2-CK2α-44 cells left untreated or treated with 1 µg/ml doxycycline for three days were co-treated with 0.1% DMSO or 0.1 µM aphidicolin for additional five days. Cells were analyzed by flow cytometry following propidium iodide staining (PI) and events were quantified and expressed in percentage.

We hypothesized that delayed entry into S-phase could be caused by defective DNA replication initiation in cells with reduced levels of CK2α. If so, we anticipated that these cells would become highly sensitive to replication stress induced in the presence of DNA replication inhibitors. To test this hypothesis, we studied cell cycle progression in response to mild replication stress in the absence or presence of 0.1 μM aphidicolin for increasing amounts of times. As shown in Fig. 3a, treatment with aphidicolin and doxycycline significantly impaired the proliferation of H9c2-CK2α-44 cells as compared to control cells or cells treated with either compounds alone. Conversely, the proliferation rate of the parental cell line was not significantly affected by the treatment with the compounds used either alone or in combination (Fig. 3b). Analysis of DNA content by flow cytometry of cells treated with doxycycline revealed a slightly but reproducible higher percentage of cells in G1-phase (Fig. 3c). Cells treated with aphidicolin showed marginally increased accumulation of cells at the G1-S border, which was expected considering the low concentration of aphidicolin employed to induce mild replication stress. Finally, incubation with both compounds resulted in accumulation of cells in both G1 and S phases (Fig. 3c). Hence, their accumulation in the early phases of the cell cycle at the expense of G2/M may explain lack of proliferation observed following induction of mild replication stress.

### Global gene expression profiling uncovers a novel role of CK2α in DNA replication initiation

**Figure 4:**
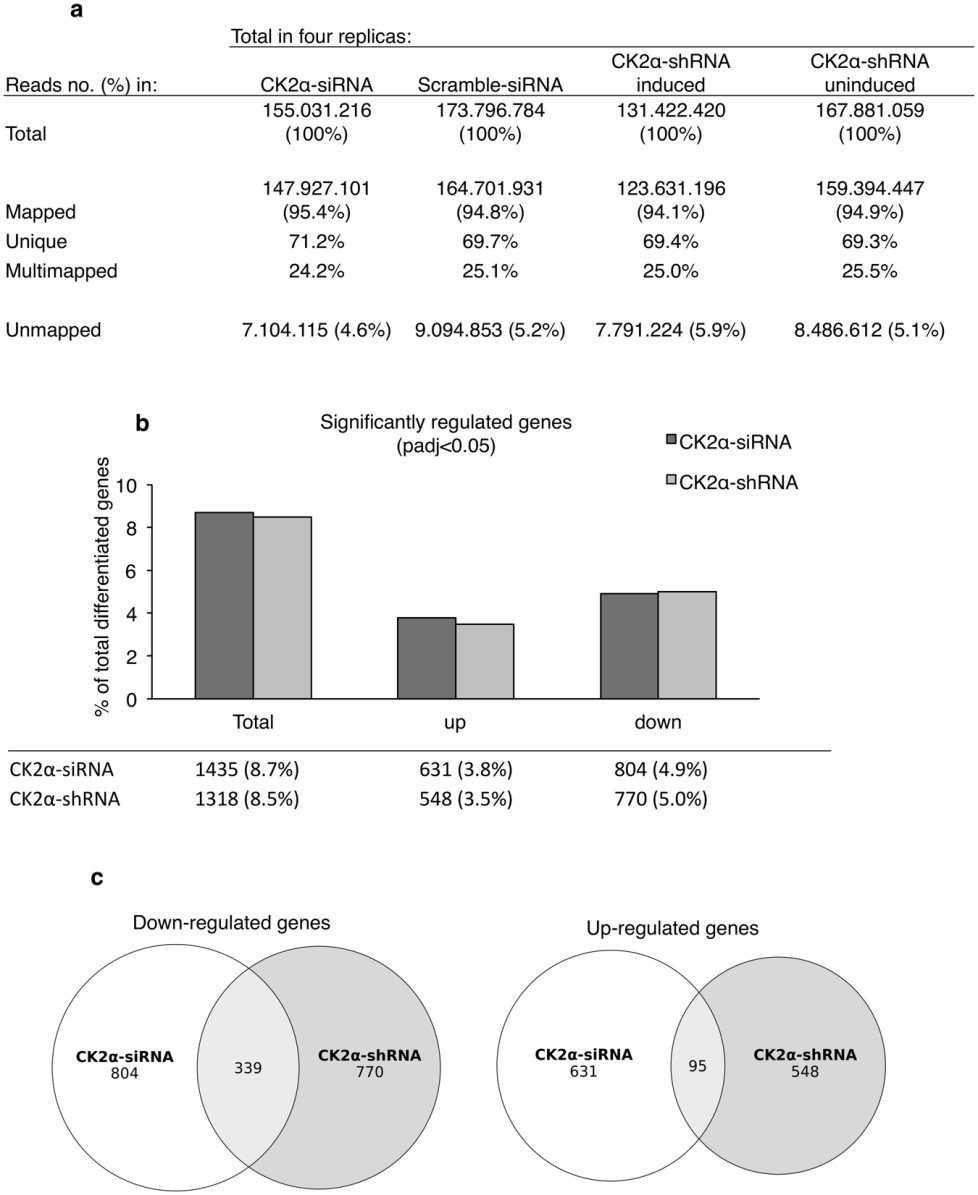
Expression analysis of RNA seq data using DESeq2. (**a**) Sequencing statistics showing total number of reads and the percentage of mapped, uniquely mapped and multi mapped reads for the CK2α-siRNA and CK2α-shRNA samples. (**b**) Bar plot showing the percentage of significantly differentiated genes for the CK2α-siRNA and CK2α-shRNA samples, respectively. The percentage of up- and down-regulated genes is also displayed. (**c**) Venn Diagrams of the overlap of the significant differentiated genes between the CK2α-siRNA and CK2α-shRNA treated cells [P-value (padj) <0.05].

To shed light on the mechanisms by which CK2α regulates cell proliferation in myoblasts, we performed a global gene expression analysis with RNA isolated from H9c2-CK2α-44 cells left untreated or incubated with doxycycline for three days. The transcriptome analysis was also carried out with the parental cell line following siRNA-mediated down-regulation of CK2α to exclude off-target effects resulting from particular sites of integration of lentiviral-based constructs into the genome of the host cells. We performed two independent experiments in duplicate obtaining, therefore, four independent replicates for each cell line and condition using the Illumina platform. From the 2 × 100 bp paired end Illumina run and after trimming of the raw reads, we generated between 131.422.420 and 173.796.784 million reads for each of the four conditions. The reads were then mapped and aligned to the rat genome version rnor6.0 using STAR version 2.5.0c. On average for the four conditions a total of 95% of the reads were mapped and around 70% of the reads were mapped uniquely (Fig. 4a). Furthermore, the quality of the data was also confirmed from proper assessment of the fastQC files, which fulfilled all quality controls. Gene expression analysis was performed using DEseq2. By comparing the log2 fold-changes of gene expression in the two cell lines (i.e. H9c2-CK2α-44 and parental cell lines), we obtained a high correlation between the generated data (Fig. 4b) indicating that gene expression modifications induced by silencing of CK2α in H9c2-CK2α-44 cells was not influenced by the integration site of the lentiviral-based construct. Interestingly, we found that the down-regulated genes were remarkably more represented within specific cellular pathways than the up-regulated genes. Of the 15542 genes identified following analysis of the H9c2-CK2α-44 cells, 1318 genes were found to be either up-regulated or down-regulated (8.5%, padj <0.05). In the case of the parental cell line, of the 16435 identified genes, 1435 were found differentially expressed (8.7%, padj <0.05). H9c2-CK2α-44 and H9c-2 cell lines shared 95 up-regulated and 339 down-regulated genes, respectively, following silencing of CK2α. From the analysis of H9c2-CK2α-44 cells, 548 genes were found up-regulated while 770 resulted down-regulated (Fig. 4c).

**Figure 5:**
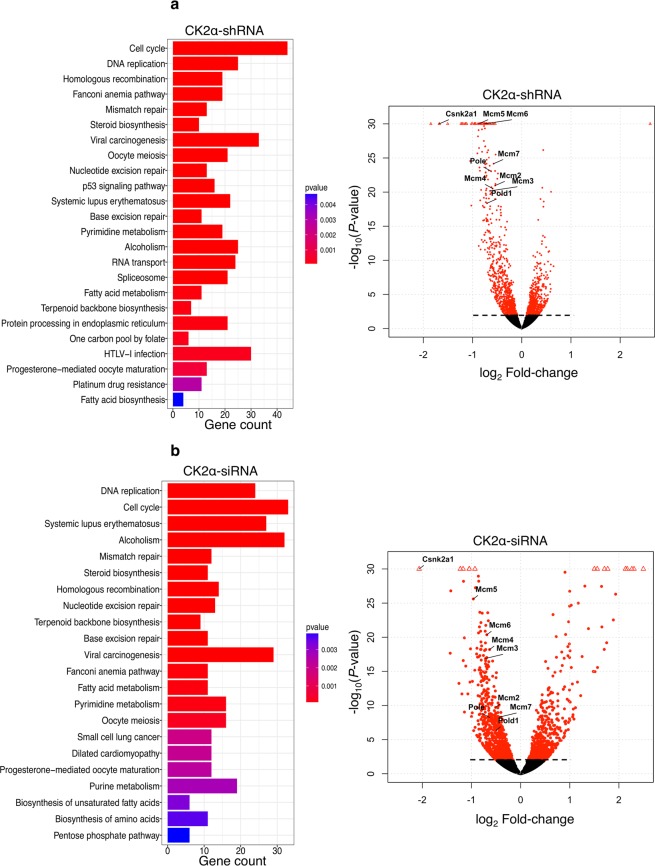
Genome-wide expression analysis reveals unique signatures in cells with reduced expression of CK2α. (**a**) KEGG pathway analysis of H9c2-CK2α-44 cells left untreated or incubated with 1 µg/ml doxycycline for three days (upper bar-plot) and (**b**) of the parental cell line (i.e. H9c-2 cell line) transfected with scramble siRNA (scr-siRNA) or siRNA directed against CK2α (CK2α-siRNA, lower bar-plot) for three days following global transcription analysis by RNA-Seq. Bar-plots show enriched pathways with respect to the number of significantly down-regulated genes within each pathway relative to controls. Panels to the right display volcano plots showing log_10_ of *P*-values against log_2_ Fold-change of gene expression. Dots above dashed line refer to genes with an adjusted *P*-value (padj) ≤0.1.

To identify the cellular pathways significantly modified in H9c2-CK2α-44 cells and in the parental cell line we performed KEGG pathway analysis on the significantly down-regulated genes (Fig. 5). Interestingly, the transcriptome analysis revealed that loss of CK2α had a substantial negative effect on the expression of genes controlling cell cycle (114/115 genes), DNA replication (33 genes) and DNA damage repair [mismatch repair (21 genes), FA pathway (46 genes), homologous recombination (25 genes), nucleotide excision repair (42 genes) and base excision repair (32 genes), Fig. 5 and Table S1]. Within the list of differentially expressed genes we found down-regulation of proliferating cell nuclear antigen (*PCNA*) gene, a number of cyclin-coding genes (*Ccna2*, *Ccnab1*, *Ccne1*, *Ccne2* and *Ccnd2*), E2F transcription factor 1 (*E2f1*) gene and the subunits 1 and 6 of the origin recognition complex (*ORC1*, *ORC6*) genes (Tables 1 and S2). Interestingly, among the most significantly down-regulated transcripts we found the minichromosome maintenance protein complex (*MCM2-7*) genes and those coding for DNA-directed polymerase epsilon, delta 1, alpha 2, delta 2, epsilon 2 (*Pole*, *Pold1*, *Pola2*, *Pold2*, *Pole2*, Table 1). Finally and as predicted, CK2α transcripts were among those most significantly down-regulated (log2 Fold-change −1.67791, padj 4.90981E-39) while CK2α’ and CK2β transcripts, were not found significantly changed (results not shown). To the best of our knowledge, a cross-talk between CK2 and MCM proteins has never been reported before. Numerous studies have shown that these proteins form pre-replication (pre-RCs) complexes, also known as “origin licensing” that allow binding of DNA polymerases and other factors to chromatin to start DNA replication during the G1-phase^29–32^. Since their reduced expression levels in cells confers hypersensitivity to replication stress^33^, we decided to further investigate them.

**Table 1:** Significantly differentially expressed genes involved in DNA replication following down-regulation of CK2α.

Ensembl_ID	Gene name	Description	CK2α-shRNA			CK2α-siRNA		
			log_2_ Fold-change	*P*-value	padj	log_2_ Fold-change	*P*-value	padj
ENSRNOG00000003703	Mcm6	minichromosome maintenance complex component 6 [Source:RGD Symbol;Acc:61967]	−0.75714	1.66E-43	1.08E-40	−0.68699	4.71E-21	1.65E-18
ENSRNOG00000014336	Mcm5	minichromosome maintenance complex component 5 [Source:RGD Symbol;Acc:1306616]	−0.87782	3.11E-32	1.12E-29	−0.9622	2.63E-26	1.60E-23
ENSRNOG00000001349	Mcm7	minichromosome maintenance complex component 7 [Source:RGD Symbol;Acc:1303018]	−0.57298	7.30E-25	1.64E-22	−0.43775	5.24E-09	3.14E-07
ENSRNOG00000037449	Polε	polymerase (DNA directed), epsilon, catalytic subunit [Source:RGD Symbol;Acc:1594540]	−0.62312	7.46E-24	1.51E-21	−0.63192	5.45E-09	3.25E-07
ENSRNOG00000016316	Mcm2	minichromosome maintenance complex component 2 [Source:RGD Symbol;Acc:1305577]	−0.54058	1.12E-21	2.01E-19	−0.5083	1.97E-10	1.53E-08
ENSRNOG00000001833	Mcm4	minichromosome maintenance complex component 4 [Source:RGD Symbol;Acc:3060]	−0.59195	3.17E-21	5.42E-19	−0.63094	6.89E-19	1.83E-16
ENSRNOG00000012543	Mcm3	minichromosome maintenance complex component 3 [Source:RGD Symbol;Acc:1305168]	−0.64377	6.62E-21	1.09E-18	−0.71333	1.32E-17	2.75E-15
ENSRNOG00000019681	Pol δ1	polymerase (DNA directed), delta 1, catalytic subunit [Source:RGD Symbol;Acc:621839]	−0.70705	3.82E-19	5.40E-17	−0.5002	4.26E-07	1.70E-05
ENSRNOG00000031993	Prim1	primase, DNA, polypeptide 1 [Source:RGD Symbol;Acc:621380]	−0.50871	4.28E-18	5.49E-16	−0.37632	1.46E-06	5.24E-05
ENSRNOG00000021264	Pcna	proliferating cell nuclear antigen [Source:RGD Symbol;Acc:3269]	−0.36135	5.93E-13	5.20E-11	−0.45828	4.36E-11	3.79E-09
ENSRNOG00000020531	Fen1	flap structure-specific endonuclease 1 [Source:RGD Symbol;Acc:621821]	−0.54247	7.06E-13	6.06E-11	−0.43085	2.10E-06	7.15E-05
ENSRNOG00000001134	Rfc5	replication factor C (activator 1) 5 [Source:RGD Symbol;Acc:1309280]	−0.50983	8.06E-11	5.52E-09	−0.28927	0.001611	0.022343
ENSRNOG00000001088	Rfc3	replication factor C (activator 1) 3 [Source:RGD Symbol;Acc:1306832]	−0.43253	1.03E-09	6.11E-08	−0.03864	0.707713	0.916089
ENSRNOG00000014193	Lig1	ligase I, DNA, ATP-dependent [Source:RGD Symbol;Acc:621424]	−0.59388	1.37E-09	8.01E-08	−0.43153	0.000422	0.007455
ENSRNOG00000003123	Rpa1	replication protein A1 [Source:RGD Symbol;Acc:1307376]	−0.3821	1.82E-08	9.02E-07	−0.30221	0.000893	0.013954
ENSRNOG00000020906	Polα2	polymerase (DNA directed), alpha 2, accessory subunit [Source:RGD Symbol;Acc:621817]	−0.41512	2.99E-08	1.43E-06	−0.43507	4.48E-06	0.000143
ENSRNOG00000001457	Rfc2	replication factor C (activator 1) 2 [Source:RGD Symbol;Acc:621198]	−0.40817	4.06E-08	1.91E-06	−0.3773	1.90E-05	0.000517
ENSRNOG00000013005	Rpa2	replication protein A2 [Source:RGD Symbol;Acc:619714]	−0.46473	6.44E-07	2.44E-05	−0.43804	0.000576	0.009691
ENSRNOG00000014098	Polδ2	polymerase (DNA directed), delta 2, accessory subunit [Source:RGD Symbol;Acc:1304954]	−0.41421	1.17E-06	4.06E-05	−0.32712	0.002745	0.034025
ENSRNOG00000001816	Rfc4	replication factor C (activator 1) 4 [Source:RGD Symbol;Acc:1310142]	−0.35667	9.75E-05	0.002146	−0.36891	0.000243	0.004763
ENSRNOG00000020700	Rnaseh2c	ribonuclease H2, subunit C [Source:RGD Symbol;Acc:2319141]	−0.37524	0.000125	0.00266	−0.37188	0.000284	0.005461
ENSRNOG00000012486	Prim2	primase, DNA, polypeptide 2 [Source:RGD Symbol;Acc:631433]	−0.30848	0.000168	0.003453	0.009264	0.920424	0.981675
ENSRNOG00000004242	Polε2	polymerase (DNA directed), epsilon 2, accessory subunit [Source:RGD Symbol;Acc:1311962]	−0.42936	0.000501	0.008671	−0.48842	0.002781	0.034413

### CK2α down-regulation correlates with decreased MCMs levels *in vitro* and *in vivo*

**Figure 6:**
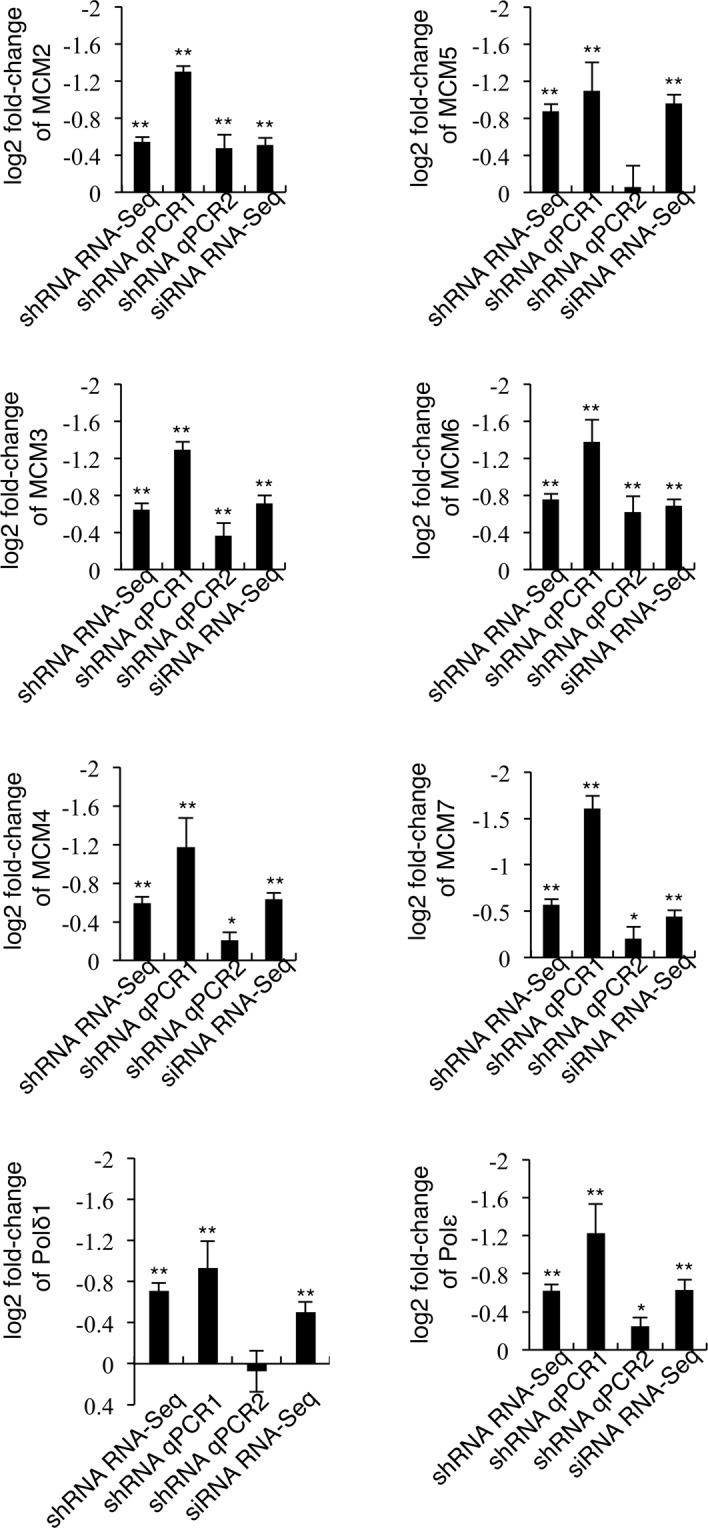
Down-regulation of CK2α leads to decreased expression of genes essential for initiation of DNA replication. H9c2-CK2α-44 cells were left untreated or treated with 1 µg/ml doxycycline for three (shRNA RNA-Seq, shRNA qPCR1) or six (shRNA qPCR2) days while the parental cell line was transfected with CK2α-siRNA for three days (siRNA RNA-Seq). Total RNA was isolated and either analyzed by Illumina TrueSeq sequencing (RNA-Seq) or used for quantitative reverse transcription PCR (RT-qPCR). Graphs show log2 Fold-change in the expression of the indicated genes from cells with reduced CK2α levels relative to control cells, respectively. As a comparison, each graph shows results obtained by both RNA-Seq and qPCR, respectively. *P < 0.01, **P < 0.001 with respect to vehicle-treated cells. RNA-Seq experiments were carried out twice in duplicates; qPCR experiments were performed three times in triplicates. Average values are shown +/− STDEV.

Using qPCR we validated the expression of eight genes (i.e. *MCM2-7*, *Polε* and *Polδ1*, Fig. 6) in cells untreated or incubated with doxycycline for three days (shRNA qPCR1). This analysis confirmed down-regulation of the aforementioned genes. qPCR analysis in cells incubated with doxycycline for six days (shRNA qPCR2) showed reduced down-regulation of six (i.e. *MCM2, 3, 4, 6, 7* and *Polε*) of the eight genes analyzed suggesting that some adaptation might have occurred.

**Figure 7:**
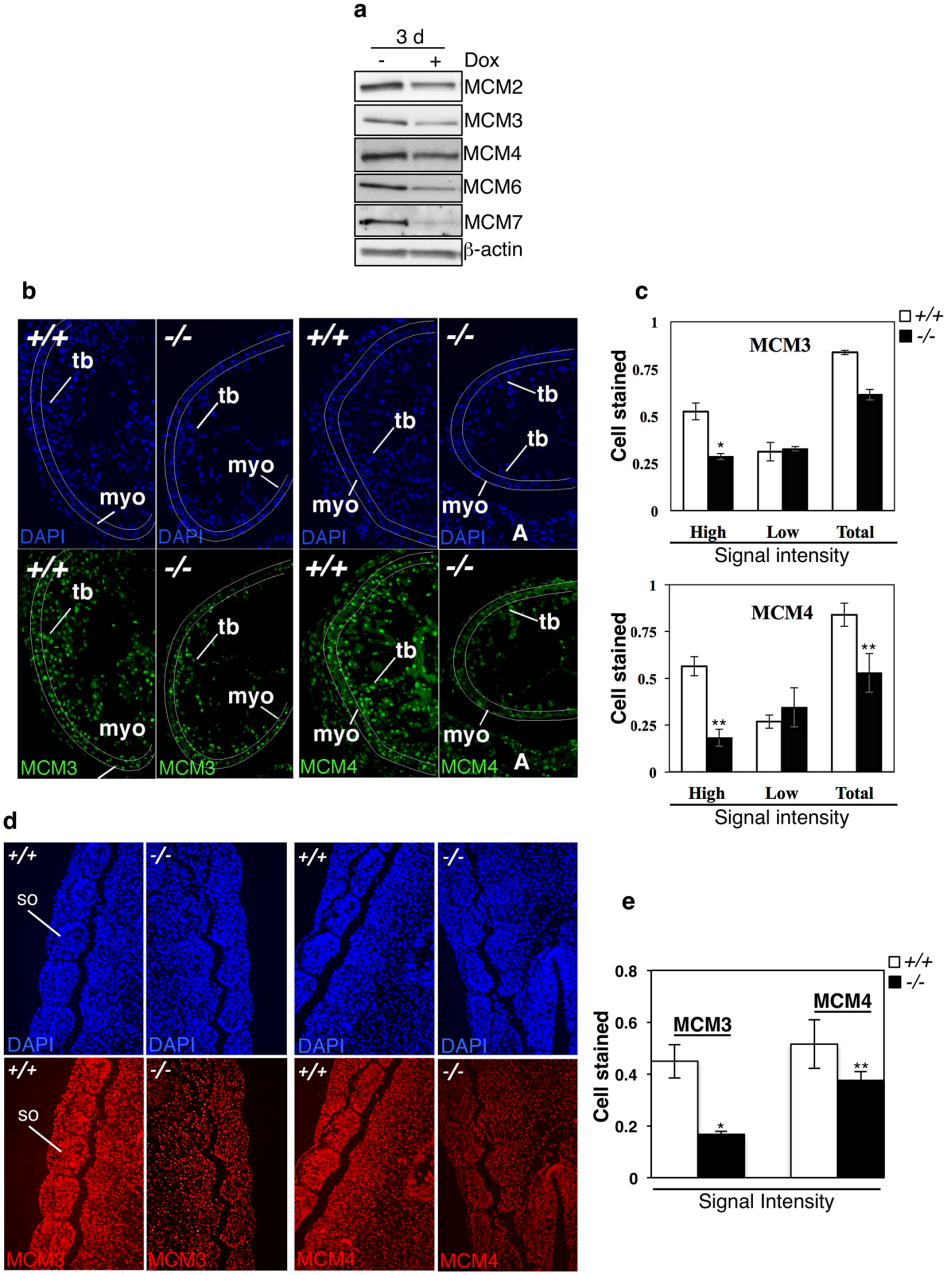
Down-regulation of gene transcripts correlates with lowered expression of the corresponding MCM proteins in CK2α-depleted cells and *CK2α−/−* mouse embryo hearts and somites. (**a**) H9c2-CK2α-44 cells were treated with 1 µg/ml doxycycline for three days and whole cell lysates were employed for the detection of MCM proteins by Western blot as indicated in the figure. β-actin detection was used as loading control. (**b**) MCM3 and MCM4 immunofluorescence staining in heart sections of *WT* (+/+) and *CK2α−/−* (−/−) embryos at E10.5 (34 somite pairs). Photographs were taken at 20x magnification. Fluorescent images were pseudo-colored and show MCM proteins staining (nuclear, green). Lines mark approximately the area where cells were counted (non-trabecular myocardium). (**c**) Bar-graph showing in percentage the ratio of MCM positive cells/total number of cells in the myocardium, and the percentage of cells showing high and low stain intensity in the myocardium, respectively. Two to three sections each from two pairs of E10.5 *WT* and *CK2α−/−* embryos were analyzed. Represented values are mean +/− STDEV. Asterisks denote statistical significance: *P ≤ 0.05, **P ≤ 0.005. Abbreviations: A (atria); myo (non-trabecular myocardium); tb (trabecular myocardium). (**d**) Immunohistochemistry analysis of whole mouse embryo somites (E10.5) showing detection of MCM3 and MCM4 (nuclear, red signal), respectively. Photos were taken at 20x magnification and pseudo-colored. (**e**) Bar-graph showing in percentage the number of MCM positive cells/total number of cells in the embryos. Three to five sections each from three pairs of E10.5 *WT* and *CK2α−/−* embryos were analyzed. Represented values are mean +/− STDEV, *P ≤ 0.0005, **P ≤ 0.05. Abbreviations: so, somite.

Next, we determined whether down-regulation of the MCM genes correlated with decreased expression levels of their coded proteins. For this, we analyzed whole lysates from cells treated with vehicle or doxycycline for three days. Results shown in Fig. 7a confirmed that silencing of CK2α results in decreased expression of MCM proteins with respect to control experiments.

*In vivo* validation of gene expression variability of two members of the MCM protein family, namely MCM3 and MCM4, was investigated in tissues of *WT* and *CK2α*-knockout embryos *i.e*.: the hearts (Fig. 7b,c) and somites at E10.5 (Fig. 7d,e). It was reported that a variety of defects in the heart at this developmental stage are probably responsible for the embryonic lethality observed in mice lacking *CK2α*^12^, therefore, we included the cardiac tissue in the analysis. In hearts, MCM3 and MCM4 specific signal was strongly detected in the cell nucleus. Negative controls for MCM4 showed some cytoplasmic background signal both in the myocardial wall and the trabeculae (Fig. S1a). We only analyzed the myocardial cells in non-trabecular myocardium, as the trabecular myocardium in *CK2α−/−* embryos is less developed than in the *WT* heart. Since MCM3 and MCM4 staining showed heterogeneous levels of fluorescence in *WT* hearts, we quantified separately cells emitting high and low fluorescence levels. Bar-graphs show a statistically significant lower number of cells emitting high intensity fluorescence in *CK2α−/−* E10.5 myocardium as compared to *WT* myocardium (Fig. 7b,c). In somites, MCM3 and MCM4 specific signal was strongly detected in the cell nucleus (Fig. 7d,e) while negative controls showed little background signal (Fig. S1b). Significant differences in the expression of MCM3 and MCM4 could be detected in *WT* and *KO* somites, respectively, suggesting that loss of CK2α affects MCM protein levels *in vivo* during early mouse development.

## Discussion

There is ample evidence showing that CK2α plays an important role in the regulation of cell division. In this study, we aimed at identifying potential novel molecular mechanisms controlling cell proliferation in non-cancerous cells mediated by CK2α. We show here for the first time that down-regulation of *CK2α* leads to a significant reduction in the expression levels of components of the MCM complex suggesting that MCM proteins could be responsible, at least in part, for the lowered proliferation rate observed in cells and *in vivo*. The minichromosome maintenance complex is a family of structurally related proteins with replicative helicase activity highly conserved from yeast to man that are required for cell proliferation, migration and invasion^34–36^. Mutations in individual MCMs have been shown to play a critical role in DNA replication initiation and result in embryonic death, growth retardation and limited fetal erythropoiesis^37–40^.

Reduced levels of CK2α resulted in decreased proliferation rate in H9c2-CK2α-44 cells (Fig. 2). Similar results were obtained when the mitotic index in *CK2α−/−* embryo hearts was compared to the one in *WT* embryos (Suppl. Fig. S2). Our data on *WT* embryonic heart proliferation fits well with previous reports showing that in *WT* embryos, OFT myocardium has the lowest proliferation rate of whole heart myocardium, and that proliferation rates diminish from E9.5 to E10 in the different heart regions^41,42^. Reduced proliferation was, however, not accompanied by a significant increase in cell death both *in vitro* (Fig. 2b) and *in vivo* (Table S3) suggesting that decreased proliferation rate may explain, at least in part, the growth defects observed in the *CK2α−/−* embryo heart.

Impaired expression of CK2α caused a reproducible slight increase in the G1/S cell population (Fig. 2). We show here for the first time that DNA replication and DNA damage pathways are significantly down-regulated in *CK2α*-silenced myoblasts. Specifically, the expression of MCMs was reduced at both mRNA and protein levels. Ibarra *et al*., demonstrated that reduced levels of the individual MCMs by RNA interference result in cells proliferating at a slower pace, accumulating in G1 phase and becoming hypersensitive to replication stress^33^. Accordingly, it is plausible that the increase in the G1/S phase population in CK2α-silenced myoblasts accompanied by enhanced sensitivity to replication stress is due to decreased MCMs expression levels.

It has been shown that mice hemizygous for the individual MCM helicases show compromised stability of the entire hexameric complex, cell proliferation defects and elevated micronuclei frequencies associated with genomic instability (GIN^43^,). The analysis of *CK2α−/−* embryo hearts revealed a drastic impairment of cell proliferation, however, it did not show increased GIN (data not shown). It is possible that a certain threshold level of expression of the MCM helicases might be necessary to cause GIN and/or that the *CK2α−/−* embryos do not live long enough to accumulate chromosomal aberrations.

A strength of this work is that we used four biological replicates for the RNA-Seq analysis, and we obtained reproducible data on proliferation rates, cell cycle aberrations and MCM expression levels both *in vivo* and with cell lines. Part of the work has been carried-out with a clonal cell line for which the risk of clonal selectivity cannot be underestimated. However, results obtained with knockout mice (Fig. 7) and siRNA-treated cells (results not shown) corroborate the findings obtained with the H9c2-CK2α-44 cells.

We observed a slight increase in CK2α’ expression levels in myoblasts depleted of CK2α (Fig. 1d). It is conceivable that the residual CK2 activity, due to the expression of CK2α’, can make up for the absence of *CK2α*. A compensatory mechanism is plausible since *CK2α* may compensate for the lack of *CK2α*’ in mice^11^. This increase in CK2α’ is similar to what has been reported in studies with mouse embryo fibroblasts (MEFs^44^), and in contrast to the lack of increase seen in the *CK2α−/−* mice^13^. This suggests that a compensatory mechanism might occur in primary cells and cell lines to preserve a certain level of CK2 kinase activity necessary for the *in vitro* survival of the cells which grow in the absence of the endogenous extracellular matrix to which cells do attach *in vivo*.

In line to what we and others have previously observed in small interfering RNA and gene knock-out studies^13,19,22,44^ cells expressing reduced amounts of CK2α also displayed reduced levels of CK2β suggesting that the contribution of the latter to the reported effects needs to be addressed in the future. Based on this, we also anticipate that a complete absence of CK2 kinase activity (i.e. double *CK2α-* and *CK2α’*-ablated mice) will have a more profound effect on cell proliferation, and experiments to test this hypothesis are under way.

Taking all these together, our data uncover a novel role of CK2α in the regulation of chromosomal DNA replication initiation, and propose that MCM helicases deregulation could be an important contributor to the observed reduction in cell proliferation in CK2α-deficient cells. We cannot, however, exclude that post-translational modifications of CK2α target proteins and/or additional genes identified in our genome wide expression analysis could contribute to the regulation of cell division.

The down-regulation of CK2α was achieved with the transduction of shRNA constructs and the transfection of small interfering RNAs, respectively. We found that the overlap of the up- or down-regulated genes in both systems was not significantly high, however, irrespective of the strategy pursued, we observed that a large number of significantly down-regulated transcripts were in common between the two systems. In this respect, although the goal is the same (i.e. the down-regulation of CK2α), the way this result is obtained differs with the two approaches. The siRNA-mediated approach involves transfection of a certain number of cells and is dependent on the variable efficiency of siRNA up-take. The lentivirus shRNA-knockdown system should, theoretically, affect all cells with respect to the silencing of a specific gene product. In the siRNA-based method, a certain amount of cells did not take up the siRNA molecules consequently; CK2α was not down-regulated in all cells. Because of this, one could not expect a perfect overlapping of the results obtained with the two systems emphasizing the importance to apply different approaches and validate significant results as we have performed in this study. Our global analysis of gene expression identified 770 significantly down-regulated genes (Fig. 4) including not only genes coding for proteins regulating DNA synthesis or DNA replication initiation but also for proteins directly regulating cell cycle phase transitions such as cyclin-dependent kinases and cyclin proteins. Furthermore, we cannot rule out that the decreased proliferation rate seen in cells depleted of CK2α might result from induction of the differentiation program. In this respect, Kankeu *et al*., carried out a proteomic analysis monitoring the changes in protein expression upon differentiation of cardiac myoblasts into cardiomyocyte-like cells and reported evidence that the proteins forming the MCM complex are significantly down-regulated in the differentiated cells^45^.

Future experiments should address the impact of miss-regulation or destabilization of the MCM complex and the mechanisms of reduced expression levels of MCMs in cells lacking CK2α. This is important, since mutations in MCMs are responsible for a number of diseases, including cancer, and malformations^29,46,47^. It will also be essential to perform rescue experiments with combinations of the MCM genes to address the importance of MCM proteins with respect to cell proliferation *in vivo* in the context of CK2α deregulation. Finally, it will also be important to generate tissue-specific CK2α knockout mice to further elucidate the function of this enzyme in different organs and define the pathways that lead to MCM deregulation. This knowledge will be pivotal for progressing our understanding of the functional specialization of protein kinase CK2α during embryonic development and in adulthood.

## Methods

### Cell culture and treatments

The myoblast cell line H9c-2 derived from embryonic rat myocardium was purchased from the American Type Culture Collection (ATCC, Rockville, MD, USA) and cultivated at 37 °C under a 5% CO_2_ atmosphere in Dulbecco’s modified Eagle’s medium (DMEM, Invitrogen, Taastrup, Denmark) supplemented with 10% fetal bovine serum (FBS, Biochrom AG, Berlin, Germany). H9c-2 cells were passaged before reaching confluence following guidelines to prevent their differentiation. Cells were treated with aphidicolin and doxycycline as indicated in the figure legends (both reagents from Sigma-Aldrich, Brøndby, Denmark). Down-regulation of protein expression was carried out by RNA interference as previously described^24^. Sets of four small interfering RNA duplexes (ON-TARGET plus SMART pools, Dharmacon, Lafayette, CO, USA) directed against *CK2α* and *CK2α’*, respectively, were used. Synchronization of cells at G0/G1 was obtained by growing them in the presence of 0.1% serum for 48 h. After that, the cell cycle was resumed by adding complete growth medium and cells were harvested at different time points as indicated in the figure.

### Gene expression silencing in H9c-2 myoblasts

The establishment of a cell line expressing shRNA for the inducible down-regulation of CK2α under the control of doxycycline was carried out with the Thermo Scientific SMARTchoice inducible shRNA kit following the manufacturer’s instructions (Thermo Scientific, Rockford, IL, USA). The SMARTchoice inducible shRNA vector consists of an inducible promoter with tetracycline response element (P_TRE3G_), activated by the Tet-On 3G protein in the presence of doxycycline (Dox). The SMARTvector contains a turbo-GFP (tGFP) reporter gene and a universal scaffold carrying shRNA directed against the *CK2α* sequence GAA.TTA.GAT.CCA.CGT.TTCA. Initially, general transduction conditions and the functional titer of viral particles were optimized, according to the manufacturer’s recommendations. Cells were treated with optimal viral titer in transduction medium containing 10% serum and 10 µg/ml polybrene for 20 h followed by addition of normal growth medium for further 24 h. Transduced cells were selected with 0.3 µg/ml puromycin for three days, re-seeded as single cells and allowed to grow until each clone contained 30 to 40 cells. The clones were analyzed by Western blot for the efficient down-regulation of CK2α following treatment with 1 µg/ml doxycycline (Dox). Analysis of green fluorescence-emitting cells was done on a FACSCalibur flow cytometer and data acquisition was carried out with Cell Quest Pro Analysis software (BD Biosciences, Franklin Lakes, New Jersey, USA). Cell pictures were taken with a Leica DMRBE fluorescence microscope equipped with a DFC 420 C camera and Leica Application Suite V 3.3.0 software (Leica Microsystem, Wetzler, Germany).

### Preparation of whole cell lysate, Western blot analysis and antibodies

Cells were harvested and further processed for Western blot analysis as previously described^19^. The following primary antibodies were employed: mouse monoclonal anti-β-actin (Sigma-Aldrich); goat polyclonal anti-MCM3 and goat polyclonal anti-MCM6 (all from Santa Cruz Biotechnology, Heidelberg, Germany); rabbit monoclonal anti-cyclin E1, rabbit monoclonal anti-MCM2, rabbit monoclonal anti-MCM4, rabbit monoclonal anti-MCM7 and rabbit polyclonal anti-P-PTEN (S380/T382/383, all from Cell Signalling Technology, MA, USA); rabbit polyclonal anti-PTEN (Upstate, Lake Placid, NY, USA). Rabbit polyclonal anti-CK2α’ was obtained by immunizing rabbits with a specific peptide sequence of human CK2α’ (i.e. SQPCADNAVLSSGTAAR). Rabbit polyclonal anti-CK2α was obtained by immunizing rabbits against the human full-length protein sequence. Mouse monoclonal anti-CK2α/α’ and mouse monoclonal CK2β were from KinaseDetect Aps, Odense, Denmark.

### Cell cycle analysis

Cell cycle analysis and determination of cell death were carried out by propidium iodide staining and flow cytometry essentially as previously described^22^. The analysis of cells was carried out with a FACSCalibur flow cytometer (BD Biosciences). Acquired data were processed by Cell Quest Pro Analysis software (BD Biosciences). For each measurement, 10,000 events were analyzed. Flow cytometry of trypsin-digested mouse embryo cells was performed as described in^48^. Cells were analyzed using a FACScan flow cytometer (BD Biosciences) and data were processed using Cell Quest Pro analysis software (BD Biosciences).

### Determination of cell proliferation

Cell proliferation was determined using the BrdU Cell proliferation Assay (Merck-Millipore, Hellerup, Denmark) following the manufacturer’s instructions. In brief, cells were incubated with BrdU for 8 h. After fixation and denaturation, cells were labeled with anti-BrdU antibody for 1 h and subsequently with goat anti-mouse IgG HRP-conjugated for 30 min. Conjugates were visualized by adding HRP substrate solution. Absorbance was measured using a spectrofotometric plate reader at dual wavelengths of 450–595 nm. Alternatively, cells were harvested by trypsinization and counted every 24 h for up to 144 h with a Neubauer improved counting chamber.

### RNA-Seq library preparation, sequencing and data analysis

Total RNA from cells was extracted using Isol-RNA lysis reagent (AH Diagnostics, Aarhus, Denmark) and chloroform followed by precipitation in isopropanol. RNA concentration, purity and integrity were analyzed applying Nanodrop and Agilent 2100 Bioanalyzer RNA 6000 nano kit (Agilent Technologies, Inc., Santa Clara, CA, USA). Only RNA with RIN ≥8.0 and a 28 s/18 s ratio of approx. 1.8 was used for sample preparation. Processing of 500 ng RNA samples for library construction was essentially carried out as described in^49^ using the TruSeq Total RNA LT Sample Prep kit, Set A (Illumina) and following the manufacturer’s instructions (Illumina TruSeq Stranded Total RNA sample preparation guide). Amplified cDNA libraries were validated in regard to size by Agilent 2100 Bioanalyzer using a DNA 1000 kit from Agilent Technologies and concentration by qPCR using the KaPa Library quantification Kits (KaPa Biosystems, Wilmington, MA, USA). 15 pM denatured libraries were loaded on the flow cell for cluster formation by bridge amplification cycles followed by paired-end 100 bp sequencing on an Illumina HiSeq1500. For data analysis, samples were first trimmed to remove TruSeq adapters using cutadapt^50^ and, subsequently, mapped to the rat genome assembly version rnor6 using STAR^51^ version 2.5.0c with junction annotation from Ensembl ver 84^52^. Subsequently, gene counts were obtained using the same Ensembl reference and HTSeq^53^, and gene expression analyzed with DESeq2^54^ using cqn^55^ derived normalization factors. Pathway analysis was performed using GAGE^56^.

### qPCR analysis

Preparation of whole RNA samples from cultured cells was carried out by phenol-chloroform extraction and subsequent silica-membrane-based purification according to the miRNeasy Mini handbook from QIAGEN (Hilden, Germany). RNA concentration and quality was determined by Nanodrop measurements and RNA integrity was assured by a bioanalyzer. cDNA was prepared using the high capacity cDNA reverse transcription kit from Life Technologies (Invitrogen). Each cDNA sample (20 ng) was used in triplicates as template for reactions using Fast Start Essential DNA Green Master (2×, Roche, Mannheim, Germany). The qPCR was performed in a Light Cycler LC480 (Roche): 1 cycle at 95 °C/5 min followed by 45 cycles at 95 °C/10 s, 60 °C/10 s, 72 °C/10 s. Primers used for qPCR (IDT, Leuven, Belgium) were designed to cover two exons thereby eliminating the risk of DNA contamination being amplified (Table S4). Threshold values were determined by the Light Cycler software (LCS480 1.5.1.62 SP1). Each qPCR assay included a no-template control. The specificity of each amplification was analyzed by melting curve analysis. Quantification cycle (Cq) was determined for each sample and was normalized (ΔC_T_) to the reference genes vimentin and chaperonin containing TCP1 subunit 2 (cct2). Reference genes were chosen based on their observed stability across conditions in the RNA-seq experiments. Fold-change of gene expression was calculated with the ΔΔC_T_ method including the equation ΔΔC_T_ = ΔC_T_ (experimental sample group) − ΔC_T_ (control group), and the fold-change was calculated based on ΔΔC_T_ with 2^−ΔΔCT^. Significance was ascertained by the two-tailed Student’s t-test.

### CK2α−/− mice and embryos isolation and processing

Mouse experiments were approved by the Boston University Medical Center Institutional Animal Care and Use Committee (IACUC) and performed in accordance with relevant guidelines and regulations. The *CK2α−/−* mice generated through a targeted mutation (*Csnk2α1*^*tm1Dcs*^^12^,) were bred, and embryos genotyped by PCR using yolk sac DNA as described in^12^. Timed mating was set by interbreeding *CK2α*+/− pairs and the morning on which a vaginal plug was observed in the female set as embryonic day E0.5. Embryos and hearts were isolated in cold PBS, and when required, hearts were excised using micro-scissors and forceps. Embryos and isolated hearts were then snap frozen on dry ice and stored at −80 °C for molecular and biochemical studies. For histological and immunological analysis, freshly dissected embryos were collected, washed in PBS and fixed at 4 °C with 4% paraformaldehyde in PBS. For histology and immunofluorescence (IF) in sections, embryos were dehydrated, embedded and sectioned^57^. For whole-mount IF (WIF), embryos were washed in PBS and stored in 70% ethanol at 4 °C. To obtain reliable results somite-matched *CK2α−/−* and WT embryos were compared in the performed analyses.

### Macroscopic and histological analyses

The cardiac phenotype was analyzed from freshly dissected heart sections, which were photographed using an Olympus SZX16. Dimensions were measured by placing lines in pictures of five *CK2α−/−* and *WT* heart pairs and analyzed using the Wilcoxon rank sum test. Heart sections from four to six somite paired *CK2α−/−* and *WT* embryos were stained with Hematoxylin-eosin (H&E) as described in^57^.

### Immunofluorescence (IF) and whole mount immunofluorescence (WIF)

WIF for phospho-histone H3/Ser10 (phH3) was performed in three somite-paired *WT* and *CK2α−/−* embryos as described in^14^. PhH3 IF staining in sections, was performed in slides with comparable sections from three somite-paired *CK2α−/−* and *WT* embryos. Slides were processed in parallel throughout the procedure as described in^14^. Fluorescent cells in the photographs were counted using ImageJ (NIH, Bethesda, MD, USA). Mitotic index was calculated as the percentage of phH3-positive cells/total number of cells (i.e. DAPI-stained cells, Invitrogen, Carlsbad, CA, USA) and were analyzed with a Wilcoxon rank sum test. Slides were mounted with ProLong Gold (Molecular Probes, Eugene, OR, USA) and photographed in a Nikon Eclipse TE-2000E/Photometrics CoolSnapHQ^2^ camera with NIS-elements software. For MCM3 and MCM4 immunofluorescence, slides with comparable sections from two somite-paired *CK2α−/−* and *WT* embryos were processed in parallel throughout all the procedures. 10% rabbit serum and 10% goat serum were used to block for MCM3 and MCM4, respectively, and no retrieving was performed for either anti-MCM antibody. Sections were incubated with antibodies overnight at 4 °C. As negative controls, in one section of each slide no primary antibody was added, and in another neither primary nor secondary antibodies were added. Photographs were taken at the same magnification and exposure time, and were analyzed using ImageJ (NIH, USA). The number of stained cells was calculated in percentage as the number of antibody-stained positive cells/ total number of cells (DAPI), and significance analyzed with the Exact Wilcoxon test. To determine the percent of high- and low-intensity stained cells, the threshold set in ImageJ was applied for both WT and KO sections. For immunofluorescence quantitation, histograms represent mean values +/− standard deviation (STDEV). Statistical analysis was performed with R Statistical package (R Foundation for Statistical Computing, Vienna, Austria; Version 2.15.1). P-values < 0.05 were considered significant.

All applied methods were performed in accordance with the relevant guidelines and regulations.

## Supplementary information

Supplementary information

## Data Availability

RNA-seq data have been deposited in the ArrayExpress database at EMBL-EBI under accession number E-MTAB-8067.
