# Supplementary material for: Down-regulation of CK2α correlates with decreased expression levels of DNA replication minichromosome maintenance protein complex (MCM) genes
Source: Sci Rep. 2019 Oct 10;9:14581. doi: 10.1038/s41598-019-51056-5 (PMC6787000; doi:10.1038/s41598-019-51056-5)

Down-regulation of CK2α correlates with decreased expression levels of DNA replication minichromosome maintenance protein complex (MCM) genes

Susanne Schaefer, Thomas K. Doktor, Sabrina B. Frederiksen, Kathleen Chea, Mirka Hlavacova, Gitte H. Bruun, Maj Rabjerg, Brage S. Andresen, Isabel Dominguez and Barbara Guerra

Supplementary Information

FIGURE LEGENDS

Figure S1. Expression pattern of MCM3 and MCM4 in mouse embryo heart and somites – negative controls. (**a**) Negative controls for MCM3 and MCM4 staining of myocardium sections from E9.5 *WT* embryos. Negative controls for MCM4 revealed some cytoplasmic background signal both in the myocardial wall and trabeculae. (**b**) Negative controls for MCM3 and MCM4 staining of somite sections from E10.5 *WT* and *KO* mouse embryos.

Figure S2. Proliferation is diminished in *CK2α-/-* embryo hearts. (**a**) Whole-mount phospho-histone H3-Ser10 (phH3) immunofluorescence in E9.5 (22-24 somite pairs) and E10 (30 somite pairs) embryos. Photographs also show bright field (BF), phH3 (red) and DAPI staining. White lines delineate heart contour. Fluorescent pictures were pseudo-colored. (**b**, **c**) phH3 immunofluorescence staining in heart sections of OFT, ventricles and atria from embryos at (b) E9.5 (22 and 24 somite pairs) and (c) E10 (34 somite pairs). Photographs show bright-field, composite phH3 (red)/DAPI (blue) staining. Fluorescent images were pseudo-colored. Scale bar (b): 50 μm, scale bar (c): 100 μm. (**d**) Bar graphs showing mitotic index at E9.5 and E10. Five to seven sections per heart region from three pairs of E9.5 *WT* and *CK2α*-/- embryos, and eight to eleven sections per heart region from three pairs of E10.5 *WT* and *CK2α*-/- embryos were analyzed. Represented values are mean mitotic index +/- STDEV. Numbers inside the bars are the total number of cells analyzed in the different heart areas. Asterisks denote statistical significance: **P*-value

≤0.05, ***P*-value ≤0.005, ****P*-value ≤0.0005. Abbreviations: A (atria); AV (atrioventricular); LV (left ventricle); OFT (outflow tract); OFTe (outflow tract endocardium); OFTm (outflow tract myocardium); RV (right ventricle).

Table S1. Significantly down-regulated KEGG pathways following CK2α silencing

KEGG pathway	CK2α-shRNA		CK2α-siRNA		No. of genes in pathway
	P-value	*q*-value	*P*-value	*q*-value	
rno03030 DNA replication	2.98E-10	7.82E-08	2.42E-06	0.000645	33
rno04110 Cell cycle	7.52E-10	9.85E-08	0.000199	0.013235	114/115
rno05322 Systemic lupus erythematosus	3.24E-07	2.83E-05	8.17E-06	0.001087	56
rno03430 Mismatch repair	1.54E-06	0.000101	0.006522	0.216869	21
rno03460 Fanconi anemia pathway	1.93E-06	0.000101	0.001038	0.046034	46
rno05034 Alcoholism	6.02E-05	0.002628	0.000407	0.021642	108/109
rno03440 Homologous recombination	8.26E-05	0.003091	0.003642	0.138394	25
rno00240 Pyrimidine metabolism	0.000167	0.005454	0.015496	0.412185	85/88
rno03420 Nucleotide excision repair	0.000238	0.006939	0.081721	0.905743	42
rno03013 RNA transport	0.00033	0.008417	0.369935	0.998532	139
rno00100 Steroid biosynthesis	0.000353	0.008417	2.84E-05	0.002515	18
rno03040 Spliceosome	0.000937	0.020455	0.959993	0.998532	124
rno03410 Base excision repair	0.001091	0.021995	0.01928	0.464968	32
rno05203 Viral carcinogenesis	0.001333	0.024948	0.363486	0.998532	176
rno03008 Ribosome biogenesis in eukaryotes	0.001586	0.0277	0.16245	0.998532	72

Table S2. Significantly differentially expressed genes involved in cell cycle regulation after CK2α down-regulation

			CK2a-shRNA			CK2a-siRNA		
Ensembl_ID	Gene name	Description	Log_2 Fold-change	*P*-value	padj	Log_2 Fold-change	*P*-value	padj
ENSRNOG00000003703	Mcm6	minichromosome maintenance complex component 6 [Source:RGD Symbol;Acc:61967]	-0.75714	1.66E-43	1.08E-40	-0.68699	4.71E-21	1.65E-18
ENSRNOG00000058539	Ccnb1	cyclin B1 [Source:RGD Symbol;Acc:2291]	-0.77392	2.60E-33	9.64E-31	-0.8011	2.27E-17	4.67E-15
ENSRNOG00000014336	Mcm5	minichromosome maintenance complex component 5 [Source:RGD Symbol;Acc:1306616]	-0.87782	3.11E-32	1.12E-29	-0.9622	2.63E-26	1.60E-23
ENSRNOG00000000632	Cdk1	cyclin-dependent kinase 1 [Source:RGD Symbol;Acc:2319]	-0.68489	6.75E-32	2.33E-29	-0.59044	8.62E-11	7.19E-09
ENSRNOG00000028415	Cdc20	cell division cycle 20 [Source:RGD Symbol;Acc:620477]	-0.80694	1.25E-26	3.19E-24	-0.75992	9.52E-15	1.31E-12
ENSRNOG00000001349	Mcm7	minichromosome maintenance complex component 7 [Source:RGD Symbol;Acc:1303018]	-0.57298	7.30E-25	1.64E-22	-0.43775	5.24E-09	3.14E-07
ENSRNOG00000010017	Wee1	WEE1 G2 checkpoint kinase [Source:RGD Symbol;Acc:1307895]	-0.85073	6.01E-24	1.25E-21	-0.7626	2.90E-10	2.17E-08
ENSRNOG00000015423	Ccna2	cyclin A2 [Source:RGD Symbol;Acc:621059]	-0.7314	6.17E-23	1.15E-20	-0.88799	4.05E-19	1.19E-16
ENSRNOG00000032778	Bub1	BUB1 mitotic checkpoint serine/threonine kinase [Source:RGD Symbol;Acc:1311707]	-0.68569	1.31E-22	2.40E-20	-0.65843	3.83E-13	4.51E-11
ENSRNOG00000016316	Mcm2	minichromosome maintenance complex component 2 [Source:RGD Symbol;Acc:1305577]	-0.54058	1.12E-21	2.01E-19	-0.5083	1.97E-10	1.53E-08
ENSRNOG00000001833	Mcm4	minichromosome maintenance complex component 4 [Source:RGD Symbol;Acc:3060]	-0.59195	3.17E-21	5.42E-19	-0.63094	6.89E-19	1.83E-16
ENSRNOG00000029055	Ttk	Ttk protein kinase [Source:RGD Symbol;Acc:1305558]	-0.72704	4.78E-21	7.99E-19	-0.78776	1.18E-13	1.45E-11
ENSRNOG00000012543	Mcm3	minichromosome maintenance complex component 3 [Source:RGD Symbol;Acc:1305168]	-0.64377	6.62E-21	1.09E-18	-0.71333	1.32E-17	2.75E-15
ENSRNOG00000014786	Ccne1	cyclin E1 [Source:RGD Symbol;Acc:2294]	-0.59781	1.24E-17	1.59E-15	-0.32873	0.000104	0.002306
ENSRNOG00000009867	Tgfb3	transforming growth factor, beta 3 [Source:RGD Symbol;Acc:3851]	-0.42481	1.29E-17	1.63E-15	0.108653	0.222859	0.595916
ENSRNOG00000007906	Bub1b	BUB1 mitotic checkpoint serine/threonine kinase B [Source:RGD Symbol;Acc:619791]	-0.63158	1.74E-13	1.64E-11	-0.63509	2.26E-07	9.71E-06
ENSRNOG00000021264	Pcna	proliferating cell nuclear antigen [Source:RGD Symbol;Acc:3269]	-0.36135	5.93E-13	5.20E-11	-0.45828	4.36E-11	3.79E-09
ENSRNOG00000005376	Mad2l1	MAD2 mitotic arrest deficient-like 1 (yeast) [Source:RGD Symbol;Acc:1310889]	-0.65225	1.06E-12	8.98E-11	-0.6286	4.70E-11	4.05E-09
ENSRNOG00000002418	Tgfb2	transforming growth factor, beta 2 [Source:RGD Symbol;Acc:70491]	-0.33439	5.32E-12	4.17E-10	0.317567	8.69E-07	3.30E-05
ENSRNOG00000027787	Cdc6	cell division cycle 6 [Source:RGD Symbol;Acc:1309157]	-0.60732	3.28E-10	2.10E-08	-0.93183	6.55E-16	1.09E-13
ENSRNOG00000008055	Ccne2	cyclin E2 [Source:RGD Symbol;Acc:1307783]	-0.57667	3.22E-09	1.79E-07	-0.43891	8.01E-05	0.001825
ENSRNOG00000016708	E2f1	E2F transcription factor 1 [Source:RGD Symbol;Acc:728892]	-0.47787	8.22E-09	4.32E-07	-0.57003	4.74E-08	2.31E-06
ENSRNOG00000007249	Cdkn1b	cyclin-dependent kinase inhibitor 1B [Source:RGD Symbol;Acc:69062]	0.40501	8.92E-09	4.64E-07	0.030421	0.833783	0.960209
ENSRNOG00000006469	Cdk2	cyclin dependent kinase 2 [Source:RGD Symbol;Acc:70486]	-0.42184	3.51E-08	1.66E-06	-0.38348	1.81E-05	0.000494
ENSRNOG00000018815	Plk1	polo-like kinase 1 [Source:RGD Symbol;Acc:3352]	-0.48325	1.82E-07	7.78E-06	-0.1957	0.29473	0.668792
ENSRNOG00000003802	Pttg1	pituitary tumor-transforming 1 [Source:RGD Symbol;Acc:68359]	-0.54822	6.27E-07	2.38E-05	-0.65373	2.77E-05	0.000724
ENSRNOG00000006921	Rbl1	retinoblastoma-like 1 [Source:RGD Symbol;Acc:1595511]	-0.45231	8.75E-07	3.16E-05	-0.6004	1.33E-07	6.02E-06
ENSRNOG00000024043	Orc6	origin recognition complex, subunit 6 [Source:RGD Symbol;Acc:1311437]	-0.41589	1.07E-06	3.80E-05	-0.14468	0.192646	0.55644

ENSRNOG00000059837	Cdkn2a	cyclin-dependent kinase inhibitor 2A [Source:RGD Symbol;Acc:2323]	-0.49048	1.81E-06	6.07E-05	0.418319	1.97E-06	6.78E-05
ENSRNOG00000050071	Cdc45	cell division cycle 45 [Source:RGD Symbol;Acc:1590928]	-0.53947	2.27E-06	7.46E-05	-0.76638	3.90E-08	1.93E-06
ENSRNOG00000029773	Atm	ATM serine/threonine kinase [Source:RGD Symbol;Acc:1593265]	-0.27703	1.61E-05	0.00044	0.045675	0.703367	0.914532
ENSRNOG00000012835	Espl1	extra spindle pole bodies like 1, separase [Source:RGD Symbol;Acc:1306266]	-0.40643	3.21E-05	0.000809	-0.32944	0.064952	0.312772
ENSRNOG00000024008	Cdc25c	cell division cycle 25C [Source:RGD Symbol;Acc:1311875]	-0.37252	5.52E-05	0.001316	-0.03979	0.723073	0.920147
ENSRNOG00000003657	Pkmyt1	protein kinase, membrane associated tyrosine/threonine 1 [Source:RGD Symbol;Acc:1305434]	-0.51486	5.89E-05	0.001394	-0.46088	0.001598	0.022235
ENSRNOG00000010027	Atr	ATR serine/threonine kinase [Source:RGD Symbol;Acc:1305796]	-0.25188	0.000148	0.00309	-0.21086	0.075174	0.338302
ENSRNOG00000029785	Cdc26	cell division cycle 26 [Source:RGD Symbol;Acc:1311342]	-0.27909	0.000215	0.004261	-0.07509	0.389727	0.750466
ENSRNOG00000059059	Skp2	S-phase kinase-associated protein 2, E3 ubiquitin protein ligase [Source:RGD Symbol;Acc:1562456]	-0.28175	0.000263	0.005023	-0.4879	1.17E-07	5.39E-06
ENSRNOG00000001265	Mad1l1	MAD1 mitotic arrest deficient-like 1 (yeast) [Source:RGD Symbol;Acc:1596881]	-0.27412	0.000295	0.005568	-0.04795	0.629858	0.886049
ENSRNOG00000008841	Orc1	origin recognition complex, subunit 1 [Source:RGD Symbol;Acc:631435]	-0.45268	0.00031	0.005803	-0.69003	1.99E-06	6.84E-05
ENSRNOG00000004420	Rad21	RAD21 cohesin complex component [Source:RGD Symbol;Acc:1594529]	-0.13776	0.001171	0.017391	-0.16192	0.154133	0.496409
ENSRNOG00000005828	Skp1	S-phase kinase-associated protein 1 [Source:RGD Symbol;Acc:1359648]	0.120802	0.001503	0.021253	0.061684	0.507349	0.826882
ENSRNOG00000055471	Ywhah	tyrosine 3-monooxygenase/tryptophan 5-monooxygenase activation protein, eta [Source:RGD Symbol;Acc:3978]	-0.18215	0.001546	0.021757	-0.10504	0.429314	0.774881
ENSRNOG00000009258	Cdk6	cyclin-dependent kinase 6 [Source:RGD Symbol;Acc:621121]	-0.22948	0.002575	0.032749	0.026596	0.768633	0.937303
ENSRNOG00000057710	Ccnd2	cyclin D2 [Source:RGD Symbol;Acc:621083]	-0.12587	0.004844	0.053968*	0.078155	0.252176	0.62761
ENSRNOG00000013090	Gadd45g	growth arrest and DNA-damage-inducible, gamma [Source:RGD Symbol;Acc:1311796]	-0.16981	0.01092	0.096437	0.343063	1.87E-05	0.000508
ENSRNOG00000047741	E2f2	E2F transcription factor 2 [Source:RGD Symbol;Acc:1593939]	-0.17979	0.129662	0.469777	-0.98085	2.71E-14	3.56E-12
ENSRNOG00000006735	Cdkn2b	cyclin-dependent kinase inhibitor 2B (p15, inhibits CDK4) [Source:RGD Symbol;Acc:2324]	-0.01014	0.919935	0.984378	0.565259	3.30E-13	3.96E-11
ENSRNOG00000006304	Mdm2	MDM2 proto-oncogene, E3 ubiquitin protein ligase [Source:RGD Symbol;Acc:1305332]	0.010698	0.822452	0.963231	0.364602	1.64E-06	5.82E-05
ENSRNOG00000019822	Gadd45b	growth arrest and DNA-damage-inducible, beta [Source:RGD Symbol;Acc:1309080]	-0.07291	0.125509	0.462163	0.326492	3.42E-05	0.000868
ENSRNOG00000005904	Cdc27	cell division cycle 27 [Source:RGD Symbol;Acc:1304921]	-0.06491	0.226996	0.616278	-0.25417	0.000291	0.005572
ENSRNOG00000012153	Rbl2	retinoblastoma-like 2 [Source:RGD Symbol;Acc:3541]	0.139961	0.048628	0.264089	-0.31504	0.000306	0.005777
ENSRNOG00000020737	Cdc25a	cell division cycle 25A [Source:RGD Symbol;Acc:621498]	-0.19788	0.016197	0.1278	-0.29728	0.002145	0.028157
ENSRNOG00000011241	Tfdp2	transcription factor Dp-2 (E2F dimerization partner 2) [Source:RGD Symbol;Acc:1305814]	-0.09599	0.462709	0.814209	-0.38345	0.003675	0.042538
ENSRNOG00000058914	Rbx1	ring-box 1, E3 ubiquitin protein ligase [Source:RGD Symbol;Acc:1308453]	-0.0612	0.299581	0.693535	0.229623	0.004098	0.046069
ENSRNOG00000008459	Anapc13	anaphase promoting complex subunit 13 [Source:RGD Symbol;Acc:1583270]	0.110976	0.202609	0.584367*	0.322565	0.005116	0.055068

*padj values close to the set threshold

Table S3. Apoptotic index in E10.5 *CK2α-/-* embryos. Four to six sections per heart region from two pairs of *WT* and *CK2α-/-* embryos were analyzed. Columns display the total number of cells analyzed in the different heart areas (DAPI+), the number of TUNEL+ cells in those areas and the calculated apoptotic index (TUNEL+/DAPI+ cells). Abbreviations: AV (atrio-ventricular); A (atria); LV (left ventricle); OFT (outflow tract); RV (right ventricle).

	**Genotype**	**Total cell number (DAPI+ cells)**	**Total TUNEL+ cells**	**Apoptotic index (%)**
OFT myocardium	+/+	1269	3	0.23
	-/-	971	0	0
OFT endocardium	+/+	1353	2	0.14
	-/-	750	1	0.13
RV	+/+	4356	2	0.04
	-/-	1738	0	0
LV	+/+	5967	7	0.11
	-/-	5510	5	0.09
AV cushion	+/+	1205	2	0.16
	-/-	1214	3	0.24
A	+/+	3336	2	0.05
	-/-	3090	5	0.16

Table S4. Sequences for forward and revers primers used in the study

Gene	Forward	Reversed
Cct2	CATCAAAGCGGCACCAAGGAAA	TTCAACAGCAAATGGGAATGCT
Vim	GGAGGAGATGAGGGAGTTGC	CCTCCTGCAATTTTTCTCGCA
Mcm2	AGAACCCAGGTGGAAAGCAC	TGGTGAAAATGGCACGGCTA
Mcm3	AACCCGTTCCAAGGATGTCT	CCAGGAGCAAGCAGAGGATT
Mcm4	GGGACAATCTGACACAGCAAT	TCAGAGTAGGCGGACAGTCT
Mcm5	AAGAAGTTTGGCATGAACCCC	CATCTGGGAGCCAGAACCATCT
Mcm6	GAGACAGAAGGCATCCGAGG	TCCCTCCAAATCGTGGGTTG
Mcm7	AGGACTATGCGATTGAGAAAGAAAA	ACTTGTTCCCGATGAGCCAG
Polδ1	AAGCTGCCGTGTTTGGAGAT	AGACCACCTTGGCATTAGCA
Polε	ACAGTTCTTGCCACAGGCAG	CAGCCACGATGTATGCTGAA

Suppl. Figure S1

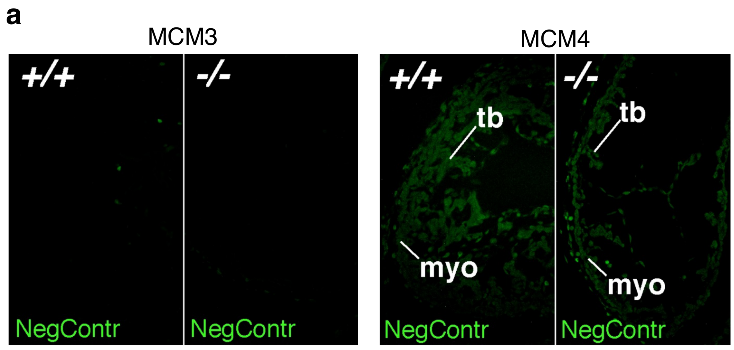

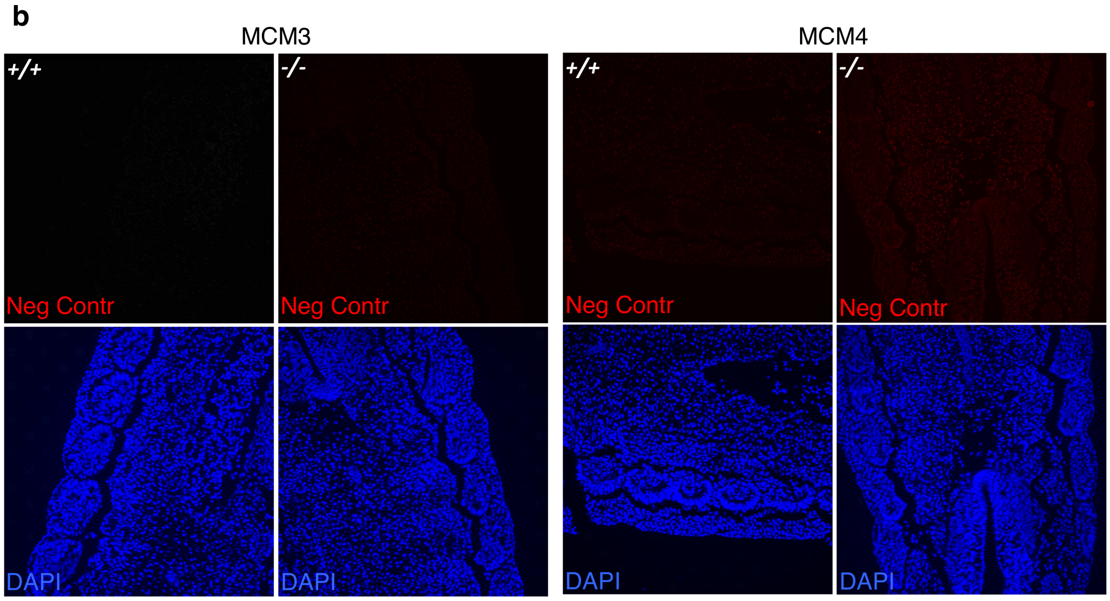

Suppl. Figure S2

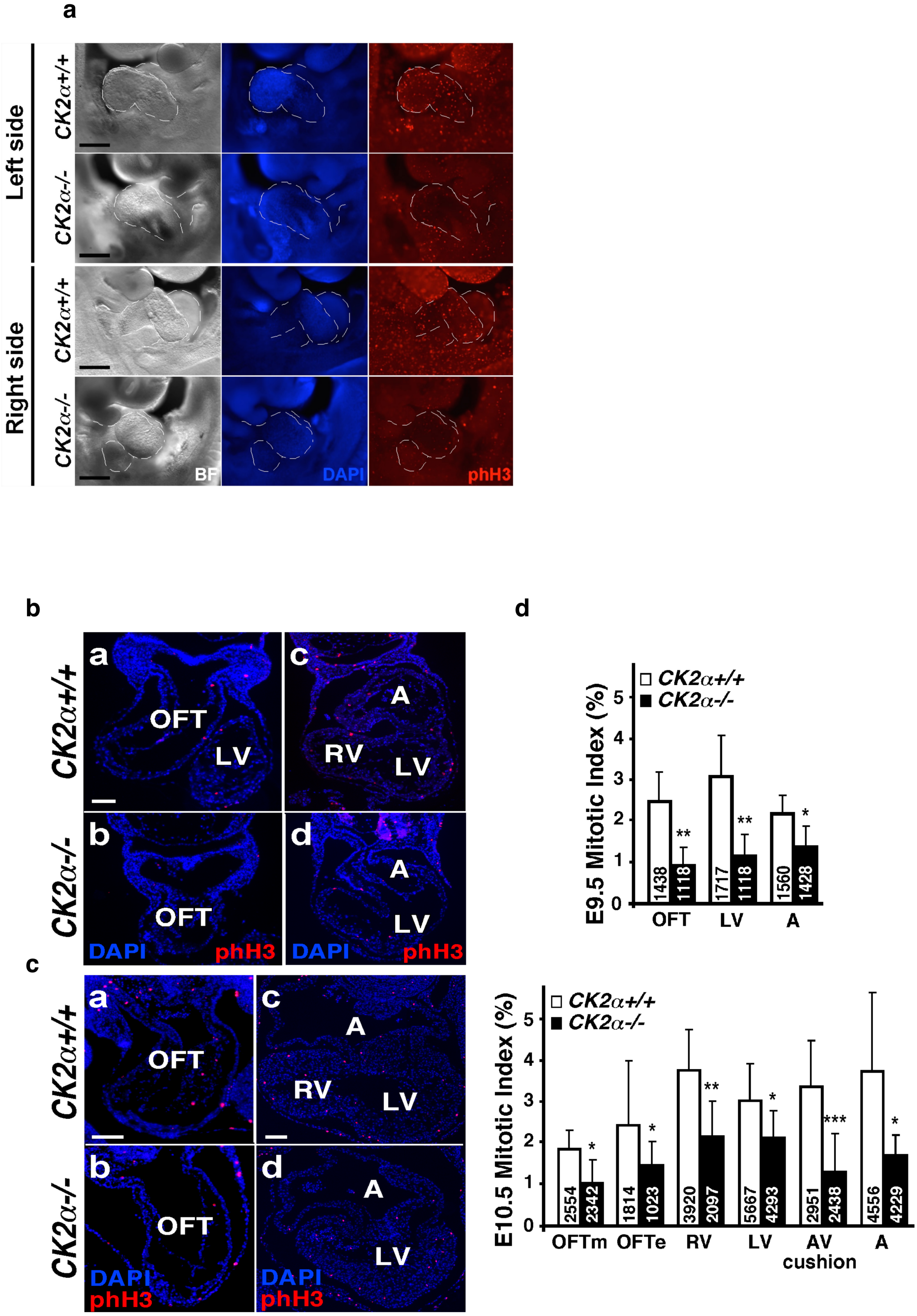

1d

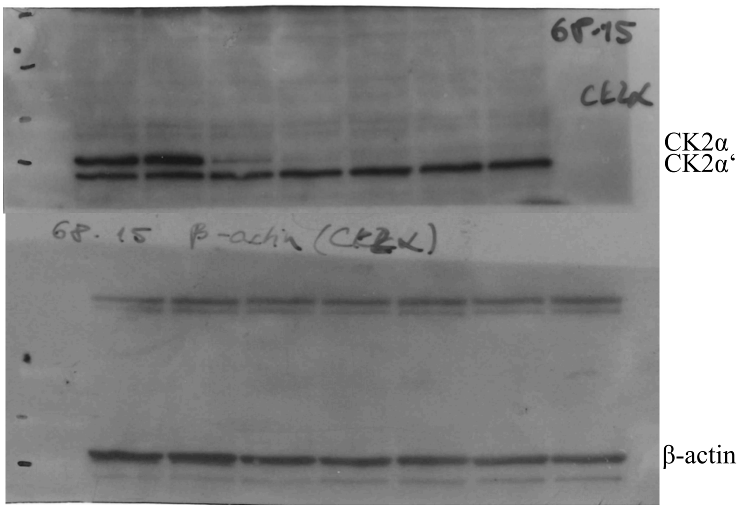
CK2α
CK2α‘
β-actin

1e

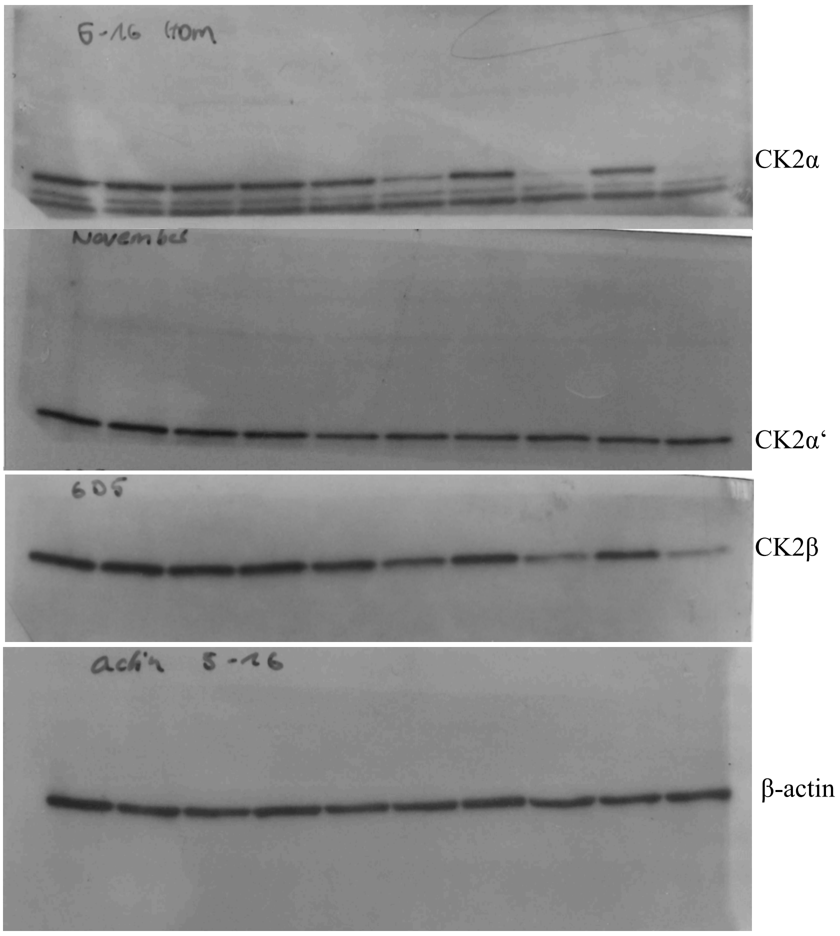
CK2α
CK2α‘
CK2β
β-actin

1f

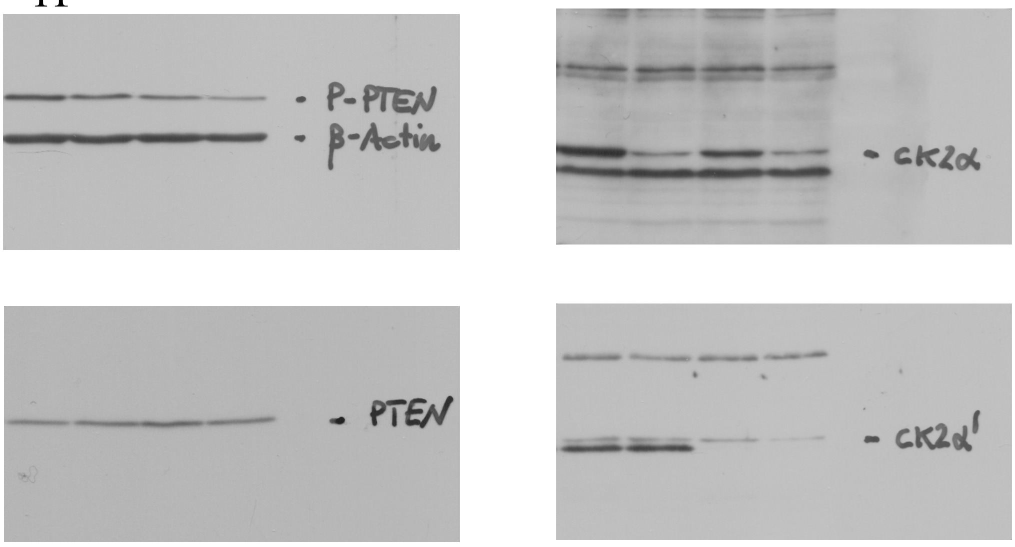
P-PTEN
β-Actin
PTEN
CK2α
CK2α'

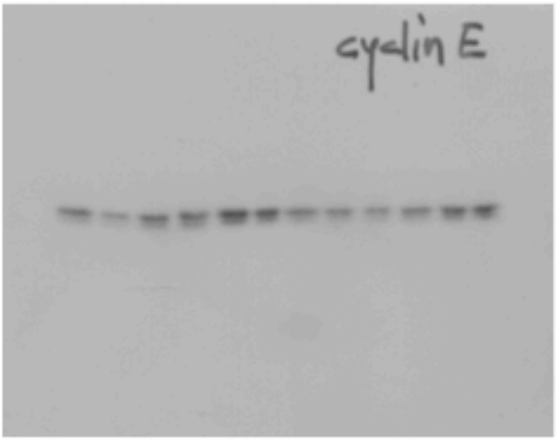
cyclin E

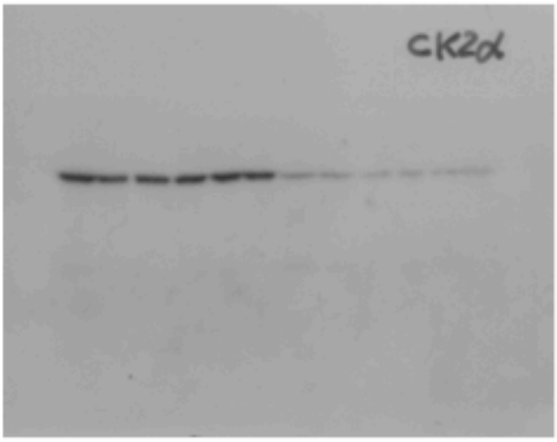
CK2α

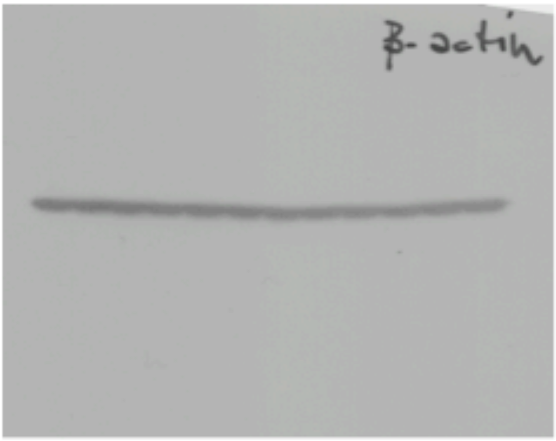
β-actin

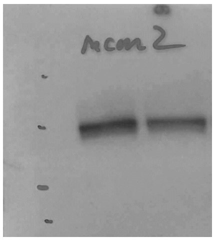
mcm2

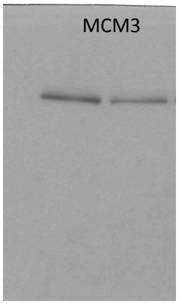
MCM3

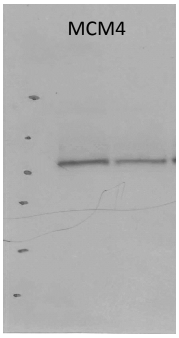
MCM4

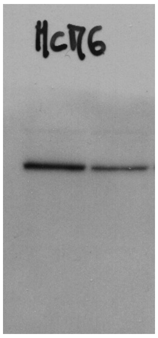
Mcm6

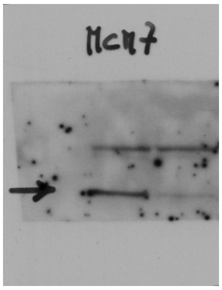
Mcm7

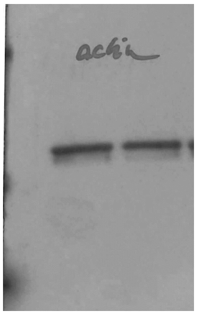

Supplement: Supplementary file 1 — Supplementary information [file 41598_2019_51056_MOESM1_ESM.pdf]
